# Overcoming resistance to immunotherapy by targeting GPR84 in myeloid-derived suppressor cells

**DOI:** 10.1038/s41392-023-01388-6

**Published:** 2023-04-28

**Authors:** Guohui Qin, Shasha Liu, Jinyan Liu, Hongwei Hu, Li Yang, Qitai Zhao, Congcong Li, Bin Zhang, Yi Zhang

**Affiliations:** 1 Biotherapy Center and Cancer Center, The First Affiliated Hospital of Zhengzhou University, Zhengzhou, Henan 450052 China; 2 Department of Medicine-Division of Hematology/Oncology, Feinberg School of Medicine Northwestern University, Chicago, IL 60611 USA; 3 School of Life Sciences, Zhengzhou University, Zhengzhou, Henan 450001 China; 4 Henan Key Laboratory for Tumor Immunology and Biotherapy, Zhengzhou, Henan 450052 China; 5 State Key Laboratory of Esophageal Cancer Prevention & Treatment, Zhengzhou University, Zhengzhou, Henan 450052 China

**Keywords:** Cancer microenvironment, Tumour immunology

## Abstract

Myeloid-derived suppressor cells (MDSCs) were found to gradually accumulate in the orthotopic esophageal cancer mouse model during tumor progression. Although the roles of MDSCs in promoting tumor growth and inhibiting immune response have been extensively explored, currently, there are still no effective means for targeting MDSCs clinically. The deficiency of specific markers of MDSCs was responsible for the limited strategy to eliminating in clinic. This study identified that GPR84 was exclusively overexpressed on MDSCs. It was further found that GPR84 was prominently expressed on MDSCs in clinical samples and tumor mouse models, which drives the immunosuppression on CD8^+^T cells by inhibiting PD-L1 degradation in lysosomes. Furthermore, G-CSF and GM-CSF were found to induce GPR84 expression through the STAT3/C/EBPβ signaling pathway. In addition, GPR84^+^MDSCs and PD-L1^+^MDSCs were highly accumulated in anti-PD-1 therapy-resistant patients with esophageal cancer, and high GPR84 signature risk was verified as a negative factor for the overall survival of patients with anti-PD-1 treatment. Finally, GPR84 antagonism combined with an anti-PD-1 antibody enhanced the antitumor responses. Therefore, targeting GPR84 enhanced anti-PD-1 efficacy in esophageal cancer and other malignant tumors. This combination therapy has the potential for tumor therapy in clinics.

## Introduction

Myeloid-derived suppressor cells (MDSCs) are a heterogeneous population of immature, immunosuppressive cells that develop in malignancies and other diseases.^1,2^ Clinical data show that MDSCs aggression is significantly and positively correlated with the clinical staging of patients with colorectal cancer, non-small cell lung cancer, and breast cancer.^3,4^ In addition, increased numbers of MDSCs interfere with sensitivity to chemotherapy and radiotherapy in patients with Hodgkin’s lymphoma and multiple myeloma.^5^ Similarly, our previous studies showed that the large accumulation of MDSCs in non-small cell lung cancer, ovarian cancer, and esophageal cancer tissues leads to treatment tolerance and poor prognosis in patients.^6–9^ Numerous studies have also demonstrated the importance of MDSCs infiltration in suppressing anti-PD-1 immunotherapy efficacy through PD-L1.^10,11^ Therefore, targeting specific markers in MDSCs is significant to remodel the immunosuppressive microenvironment and improve the efficacy of anti-PD-1 therapies.

Free fatty acid receptors (FFARs), members of the G protein-coupled receptor (GPCR) superfamily, mediate cellular responses to a variety of extracellular signals with seven-transmembrane-domain receptors.^12^ These receptors are considered to be promising targets for drug development. GPR84, also known as EX33, binds with natural ligands including caprylic acid, capric acid, and other medium-chain fatty acids (MCFAs), to reduce the accumulation of intracellular cAMP and promote Ca^2+^ influx, thereby interfering with the differentiation and function of bone marrow cells and leukocytes.^13^ Investigations have shown the key role of GPR84 in Alzheimer’s disease,^14^ diabetic nephropathy,^15^ and inflammatory bowel disease.^16^ GLPG1205, a selective GPR84 inhibitor identified by Galapagos NV, has been shown to significantly reduce the progression of inflammatory bowel disease induced by dextran sodium sulfate (DSS).^17^

In addition to inflammation, increased expression of GPR84 in stem cells has been shown to accelerate the progression of leukemia by inhibiting Ms4a3 and promoting the expression of β-catenin to enhance the proliferation and migration of leukemia cells.^18^ Although studies have demonstrated that GPR84 plays an important role in the inflammatory response, its effects on the progression of solid tumors and their underlying mechanisms are unclear.^19^

Here, we identified GPR84, predominantly expressed on MDSCs, as a promoter of immunosuppression that reduces the response to immune checkpoint blockade therapies for solid tumors in both mouse and clinical samples. Mechanismly, GPR84 drives the suppressive effect of MDSCs on CD8^+^ T cells by inhibiting PD-L1 degradation in lysosomes. Furthermore, G-CSF and GM-CSF were found to induce GPR84 expression through the STAT3/C/EBPβ signaling pathway. Inhibition of GPR84 could liberate CD8^+^ T cell dysfunction and enhance the efficacy of anti-PD-1 therapy in mouse models. These data revealed that GPR84 contributed to MDSCs suppressive function and could be a promising target to enhance anti-PD-1 therapy efficiency.

## Results

### GPR84 promotes immunosuppression in orthotopic esophageal cancer model

An orthotopic esophageal cancer mouse model was constructed using mutagen 4-nitroquinoline nitrogen oxide (4-NQO). The body weights of mice in the 4-NQO group gradually decreased compared with those in the control group (Supplementary Fig. 1a). Pathological results showed that normal mucosa developed into atypical hyperplasia and eventually to esophageal carcinoma on day 113 and 162 (Supplementary Fig. 1b). During the development of esophageal carcinoma, MDSCs frequency gradually increased, while CD8^+^ T cells and nature killer (NK) cells decreased (Supplementary Fig c). More importantly, it was found that the elevated MDSCs were mainly G-MDSCs in the mouse model of orthotopic esophageal carcinoma (Supplementary Fig 1d). The results were consistent with our previous study on clinical samples.^6^ Immunofluorescence was performed to identify the infiltration and location of MDSCs and CD8^+^ T cells in the microenvironment of esophageal carcinoma. The results showed that the total number of MDSCs and the number per mm^2^ increased significantly with the progression of esophageal carcinoma, but the number of CD8^+^ T cells decreased (Supplementary Fig. 1e–g). And MDSCs is closer to CD8^+^ T cells (Supplementary Fig. 1h), which indicates that the increase of MDSCs aggregation is significant to the formation of immunosuppressive microenvironment in advanced esophageal cancer.

**Fig. 1:**
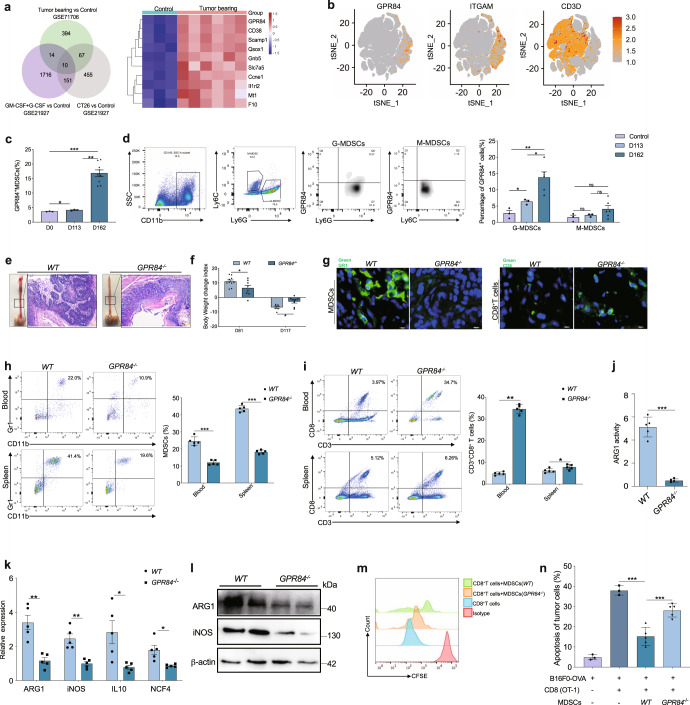
GPR84 deficiency block MDSCs immunosuppression and prevent esophageal cancer. **a** Venn analysis showed the upregulatory molecule in tumor-associated MDSCs and the top ten genes in esophageal cancer were shown in heatmap. **b** t-Distributed stochastic neighbor embedding (t-SNE) visualization of GPR84, ITGAM, and CD3D. **c** Comparison analysis of GPR84^+^ MDSC frequency in esophageal tumors in normal control (Control, *n* = 3), early stage (D113, *n* = 3) and late stage of esophageal cancer (D162, *n* = 9) after 4-NQO stimulation by flow cytometry. **d** The expression of GPR84 on M-MDSCs (Control, *n* = 3; D113, *n* = 3; D162, *n* = 6) and G-MDSCs (Control, *n* = 3; D113, *n* = 3; D162, *n* = 6) were performed in late-stage esophageal cancer (D162) by flow cytometry. **e** Gross and microscopic specimens of 4-NQO induced esophageal cancer model in *WT* and *GPR84*^*−/−*^ mice. **f** Body weight was measured on days 81 and 117 after 4-NQO stimulation and the change index was calculated to determine the progression of esophageal cancer in *WT* and *GPR84*^*-/-*^mice. **g** Immunofluorescence analysis demonstrates the infiltration of MDSCs and CD8^+^ T cells in esophageal cancer tissues. **h**, **i** Flow cytometry has been performed to analyze the accumulation of MDSCs and CD8^+^ T cells in blood and spleen. **j**, **k** MDSCs was purified using isolation kit (Miltenyi); qRT-PCR and western blotting have been performed to investigate the different expression of immunosuppressive molecule in MDSCs between *WT* and *GPR84*^*−/−*^ mice. **l** ARG 1 activity in MDSCs from *WT* and *GPR84*^*−/−*^ mice was detected by Arginase Activity Assay Kit. **m** Coincubation system was performed to investigate the immunosuppression of MDSCs on CD8^+^ T cells proliferation. **n** The antitumor activity of CD8^+^ T cells cocultured with MDSCs from *WT* and *GPR84*^*−/−*^ esophageal cancer mice were analyzed using flow cytometry. Data are represented as mean ± SEM. *p < 0.05, **p < 0.01, ***p < 0.001

To identify the one inducing the aggregation and function of MDSCs in cancer, we downloaded the mRNA sequence data of MDSCs from GEO database, and significantly differential gene clustering analysis were first performed (Supplementary Fig. 2). Venn analysis was performed on the highly expressed genes of MDSCs in each group, and 10 genes that were commonly elevated. Among them, GPR84 had the highest expression levels in esophageal carcinoma (Fig. 1a). To further verify the GPR84 expression and distribution, we downloaded the single-cell sequencing data of esophageal cancer (GSE145370) and found that the distribution of GPR84 and CD11b were highly consistent and it barely expressed on T cells (Fig. 1b). Furthermore, it was found that the percentage of GPR84^+^ MDSCs clearly increased during the development of esophageal cancer following 4-NQO challenge (Fig. 1c). And in MDSCs subsets, there is an obviously high expression of GPR84 in G-MDSC rather than M-MDSCs (Fig. 1d). Then the analysis of spleen cells from esophageal cancer mice showed that GPR84 is dominantly expressed on MDSCs, but barely expressed on B cells, T cells, and NK cells (Supplementary Fig. 3a). Detections of spleen samples derived from Lewis lung carcinoma (LLC) and B16F0 models also had given the same evidence that GPR84 is highly expressed in MDSC than other immune cells (Supplementary Fig. 3b).

*GPR84* deficient (*GPR84*^*−/−*^) mice were generated to evaluate their role in tumor development. Previous reports showed that GPR84 knockout had no effect on normal myelopoiesis. In this study, we verified that GPR84 knockout did not change the proportions of MDSCs, macrophages, B cells, NK cells, CD4^+^T cells and CD8^+^ T cells in tumor-free mice (Supplementary Fig. 4a–c). Moreover, the expression of functional molecule derived from CD4^+^ T cells or CD8^+^ T cells in *GPR84*^*−/−*^ tumor-free mice was not different from that of *WT* mice (Supplementary Fig. 4d and e).

In the orthotopic esophageal cancer model, GPR84 deficiency blocked tumor progression and inhibited body-weight loss (Fig. 1e, f). Immunofluorescence results demonstrated that *GPR84* deficiency blocked MDSCs infiltration and restored CD8^+^ T cells aggression in esophageal cancer microenvironment (Fig. 1g). Moreover, flow cytometry results showed that MDSCs were reduced and CD8^+^ T cells increased in blood and spleen of *GPR84*^*−/−*^ esophageal cancer mice (Fig. 1h, i). Then we also found that immunosuppressive molecules were inhibited in MDSCs derived from *GPR84*^*−/−*^ esophageal cancer mice (Fig. 1j, k). Also, the activity of ARG 1 was suppressed in *GPR84*^*−/−*^ MDSCs (Fig. 1l). Then, CD8^+^ T cells obtained from OT-1 transgenic mice were cocultured with B16F0-OVA cells to further verify MDSCs immunosuppressive function. The coincubation system verified that *GPR84*^*−/−*^ MDSCs lacked the capability of inhibiting the proliferation and functions of CD8^+^ T cells (Fig. 1m, n).

### GPR84 contributes to tumor progression dependent on MDSCs immunosuppressive activity in pan cancer

**Fig. 2:**
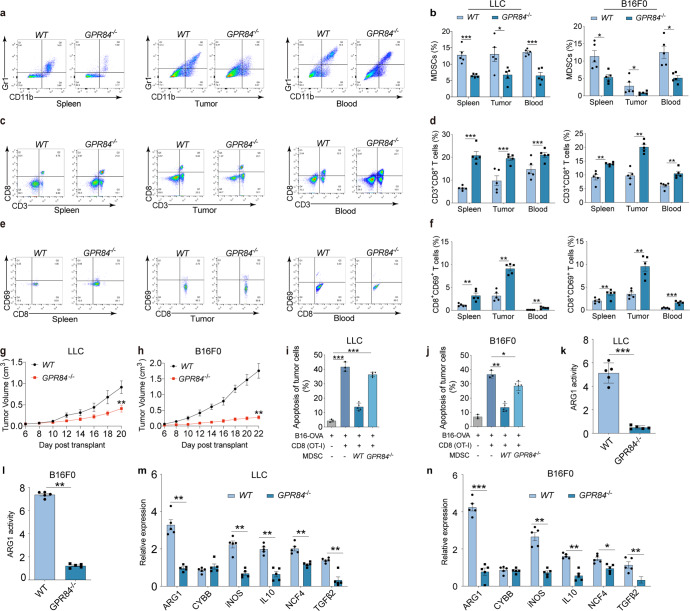
GPR84 contributes to lung cancer and melanoma progression dependent on MDSCs immunosuppression. Percentages of MDSCs (**a**, **b**), CD3^+^CD8^+^ T cells (**c**, **d**) and CD8^+^CD69^+^ T cells (**e**, **f**) in spleen, tumor tissue, and peripheral blood of LLC-cell and B16F0-cell-injected *WT* and *GPR84*^*−/−*^ mice were analyzed by flow cytometry. **g**, **h** Tumor volumes of *WT* and *GPR84*^*−/−*^ mice were measured after injection of LLC cells and B16F0 cells. **i**, **j** The specific antitumor activity of CD8^+^ T cells cocultured with MDSCs from *WT* and *GPR84*^*−/−*^ tumor-bearing mice was analyzed using flow cytometry. **k**, **l** Arginase-1 activity in MDSCs from *WT* and *GPR84*^*−/−*^ mice given LLC cells and B16F0 cells injection were analyzed. **m**, **n** The relative expression of immunosuppressive molecules (ARG 1, CYBB, iNOS, IL-10, NCF4, and TGF-β2) in MDSCs from *WT* and *GPR84*^*−/−*^ mice were analyzed using qPCR. Data are represented as mean ± SEM. *p < 0.05, **p < 0.01, ***p < 0.001

To explore whether the effect of GPR84 is specific in orthotopic esophageal cancer, subcutaneous transplantation tumor models of lung cancer (LLC) and melanoma (B16F0) were constructed. Flow cytometry revealed that the proportions of MDSCs were significantly reduced in the spleen, tumor, and blood of *GPR84*^*−/−*^ tumor-bearing mice compared to the wild-type (WT) group (Fig. 2a, b), whereas CD8^+^ T cells were remarkably increased (Fig. 2c, d) in *GPR84*^*−/−*^ tumor-bearing group. In addition, the proportions of activated CD8^+^CD69^+^ T cells were clearly increased in the tumor tissues, blood, and spleen of *GPR84*^*−/−*^ mice (Fig. 2e, f). And it showed that *GPR84* deletion markedly reduced tumor growth in lung cancer and melanoma tumor models (Fig. 2g, h). The immunosuppressive ability of MDSCs from *GPR84*^*−/−*^ mice on CD8^+^ T cell antitumor response was remarkably inhibited compared with those cells from *WT* mice (Fig. 2i, j). Furthermore, the expression or activity of MDSCs-associated molecules (ARG1, CYBB, iNOS, IL-10, NCF4, and TGF-β2) were significantly decreased from *GPR84*^*−/−*^ mice (Fig. 2k–n) in both LLC and B16F0 tumor models.

**Fig. 3:**
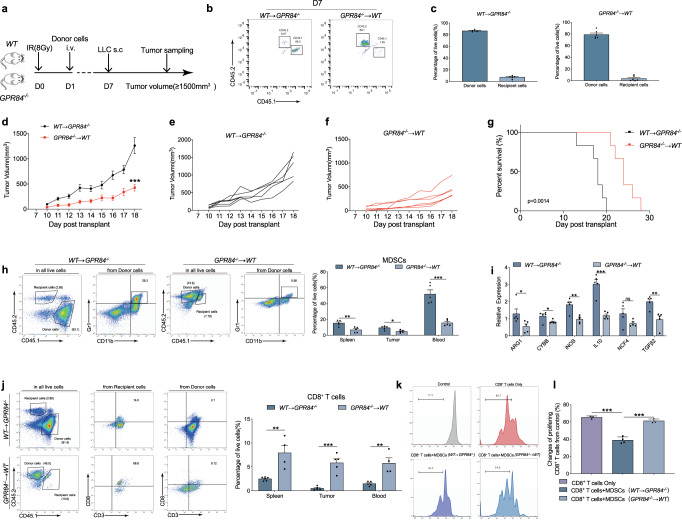
GPR84^−/−^ myeloid cells contribute to inhibit tumor progression by reducing MDSCs. **a** *WT* (CD45.1) and *GPR84*^*−/−*^ (CD45.2) mice were received 8 Gy (4 Gy*2) of radiation. Bone marrow cells collected from *GPR84*^*−/−*^ and *WT* tumor-free mice were injected intravenously to *WT* and *GPR84*^*−/−*^ mice respectively on the first day after radiation. Subcutaneous injection of LLC cells was performed on the seventh day after radiation. And the mice were sacrificed for further detection when the tumor volume reached about 1500 mm^3^. **b**, **c** The efficiency of radiation on inhibiting normal myelopoiesis was investigated on Day7 by flow cytometry (*n* = 5 per group). **d**–**f** From the tenth day after radiation, the tumor volume was recorded every day. **g** When the tumor volume was progressed to about 1500 mm^3^, the LLC mice will be sacrificed. And the overall survival was analyzed by Kaplan–Meier method. **h** Percentages of MDSCs in spleen, tumor tissue, and peripheral blood of *WT* → *GPR84*^*−/−*^ and *GPR84*^*-/*^ → *WT* group were analyzed by flow cytometry. **i** The expression of immunosuppressive molecules (ARG1, CYBB, iNOS, IL-10, NCF4, and TGF-β2) in MDSCs from the spleen were analyzed using qPCR. **j** Proportions of CD3^+^CD8^+^ T cells in spleen, tumor tissue, and peripheral blood of *WT* → *GPR84*^*−/−*^ and *GPR84*^*−/−*^ → *WT* group were analyzed by flow cytometry. **k**, **l** The effect of different MDSCs on the proliferation of CD8^+^ T cells were performed by flow cytometry. (MDSCs: CD8^+^ T cells was 4:1). Data are represented as mean ± SEM. *p < 0.05, **p < 0.01, ***p < 0.001

In order to further verify that GPR84 promotes tumor progression mainly by increasing the number and function of MDSCs, the adoptive transferred of myeloid cell model was used. *WT* (CD45.1) and *GPR84*^*−/−*^ (CD45.2) mice were received 4 Gy*2 doses of radiation. On the first day after radiation, bone marrow cells derived from *GPR84*^*−/−*^ and *WT* mice were injected intravenously respectively, that is, they were divided into two groups: *WT*→*GPR84*^*−/−*^ and *GPR84*^*−/−*^→*WT*. Subcutaneous injection of LLC cells was performed on the seventh day. After that, the growth of the tumor was recorded, and the mice were sacrificed for further detection when the tumor volume reached about 1500 mm^3^ (Fig. 3a). On the 7th day after irradiation, the percentage of donor cells in blood was more than 85% (Fig. 3b, c). Importantly, the tumor growth curve showed significant suppression in *GPR84*^*−/−*^→*WT* group (Fig. 3d–f). And it also demonstrated that the overall survival of mice in *GPR84*^*−/−*^→*WT* group were significantly increased (Fig. 3g). More importantly, the flow cytometry results showed that the proportion of MDSCs in spleen, tumor tissues and blood in *GPR84*^*−/−*^→*WT* group were decreased (Fig. 3h). And the expression of immunosuppressive makers in MDSCs purified from the spleen of *GPR84*^*−/−*^→*WT* mice were significantly reduced (Fig. 3i). However, the aggression of CD8^+^T cells increased significantly in *GPR84*^*−/−*^→*WT* group (Fig. 3j). Coculture experiment further proved that the inhibitory effect of MDSCs from *GPR84*^*−/−*^→*WT* mice on the proliferation of CD8^+^ T cells was significantly blockade (Fig. 3k, l). This adoptive transfer of myeloid cells models further verified that GPR84 deficiency dominantly inhibited MDSCs accumulation and function to reverse the immunosuppressive tumor microenvironment and enhance the prognosis of malignant tumors.

### GPR84 is negatively correlated with the prognosis of esophageal cancer

**Fig. 4:**
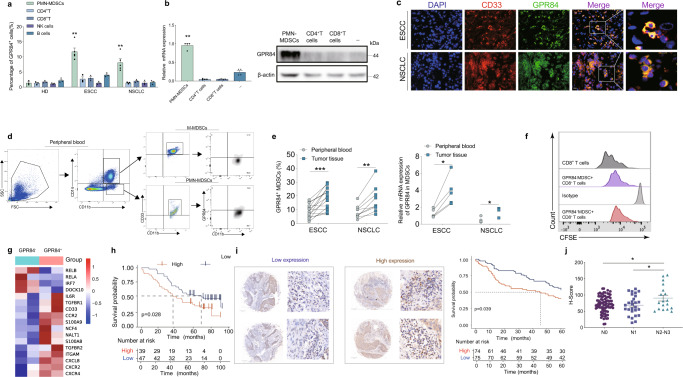
GPR84 expression patterns in clinic. **a** CD4^+^ T, CD8^+^ T, NK, and B cells from ESCC and NSCLC analyzed by flow cytometry (HD, *n* = 3; ESCC, *n* = 5; NSCLC, *n* = 5). **b** mRNA and protein expression of GPR84 in purified MDSCs, CD4^+^ T, and CD8^+^ T cells from ESCC tissues analyzed by qPCR and western blotting, respectively. **c** Evaluation of CD33 (red) and GPR84 (green) expression in cells from ESCC and NSCLC tissues using immunofluorescence; DAPI (blue). **d** Representative images showed the expression of GPR84 in M-MDSCs and PMN-MDSCs respectively. **e** Comparison of GPR84^+^ MDSC percentages and GPR84 mRNA expression in MDSCs between esophageal squamous cell cancer (ESCC) and NSCLC tissues and peripheral blood by flow cytometry. **f** Proliferation of CD8^+^ T cells cocultured with GPR84^+^ MDSCs and GPR84^-^ MDSCs derived from esophageal cancer patients. **g** Cluster analysis showed the significantly different expression genes between GPR84^+^ MDSCs and GPR84^-^ MDSCs detected by RNA sequencing. **h** Overall survival of patients with high or low GPR84 expression based on Kaplan–Meier analysis of esophageal cancer tissues from The Cancer Genome Atlas (TCGA). **i** Immunohistochemical analysis of GPR84 expression in esophageal tumors, followed by estimation of overall survival of patients with high or low GPR84 expression by Kaplan–Meier analysis. **j** GPR84 expression in esophageal cancer tissues with or without lymph node metastasis. (N0: non-lymph node metastasis, N1: metastatic lymph node ≤ 2, N2: metastatic lymph node ≥ 3). Data are represented as mean ± SEM. *p < 0.05, **p < 0.01, ***p < 0.001

Clinically, the proportion of GPR84^+^ MDSCs was significantly higher than those of other GPR84^+^ immune cell subpopulations, including CD4^+^ T cells, CD8^+^ T cells, NK cells, and B cells. Notably, the GPR84^+^ cell frequencies in peripheral blood from patients with esophageal squamous cell cancer (ESCC) and non-small cell lung cancer (NSCLC) were significantly higher than those in samples from healthy controls (Fig. 4a). Moreover, the expression levels of GPR84 were remarkably higher in purified MDSCs than in purified CD4^+^ and CD8^+^ T cells from esophageal cancer tissues (Fig. 4b), suggesting that GPR84 was specifically highly expressed on MDSCs in patients with ESCC. Immunofluorescence investigations confirmed that GPR84 was highly expressed in the CD33^+^ MDSCs of cancer tissues (Fig. 4c). These findings suggest that GPR84 is predominantly expressed on MDSCs in the tumor microenvironment.

Flow cytometric analysis of peripheral blood and tumor tissue from ESCC patients demonstrated that GPR84 was highly expressed in polymorphonuclear-MDSCs (PMN-MDSCs) than monocytic-MDSCs (M-MDSCs) (Fig. 4d). In addition, GPR84^+^ MDSCs were more prevalent in samples from ESCC and NSCLC tissues compared to peripheral blood (Fig. 4e). Functionally, it was found that GPR84^+^ MDSCs isolated from ESCC tissues inhibited the proliferation of CD8^+^ T cells (Fig. 4f). And mRNA sequencing results demonstrated GPR84^+^ MDSCs isolated from ESCC tissues were highly expressed immunosuppressive molecules (Fig. 4g). Overall survival analysis in patients with esophageal cancer from The Cancer Genome Atlas (TCGA) database also showed that patients with high GPR84 expression had a worse prognosis than did those with low GPR84 expression (Fig. 4h). Our investigations of tumor tissues from ESCC patients by immunohistochemical demonstrated that increased expression of GPR84 significantly shortened the patients’ overall survival (Fig. 4i). Patients with lymph-node metastasis showed higher GPR84 expression in tumor tissues than non-metastatic patients (Fig. 4j). Taken together, these data suggest that GPR84 is negatively correlated with the prognosis of esophageal cancer and it enhanced MDSCs immunosuppressive characteristics in ESCC microenvironment.

### Blockade of GPR84 inhibits tumor progression by remodeling the immunosuppressive microenvironment

**Fig. 5:**
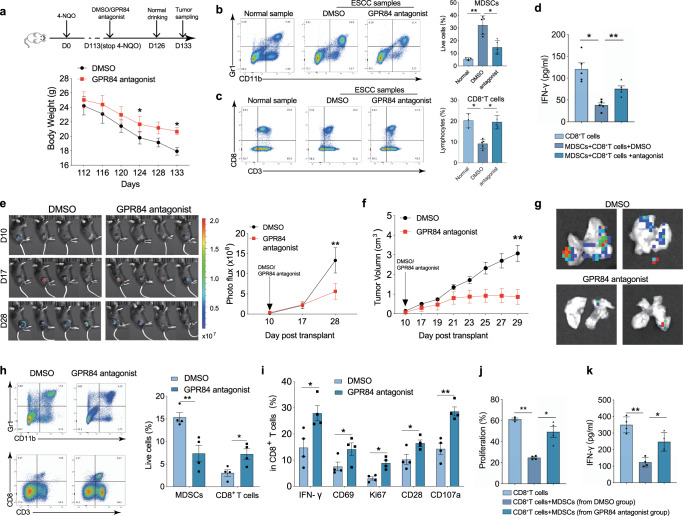
Blockade of GPR84 inhibits tumor progression by activating CD8^+^ T cells. **a** Body weights of mice treated with GPR84 antagonist or dimethyl sulfoxide (DMSO) measured from days 112 to 133 after 4-NQO stimulation. Percentages of MDSCs (**b**) and CD8^+^ T cells (**c**) were analyzed in esophageal carcinoma produced in response to 4-NQO stimulation before and after treatment with GPR84 antagonist. **d** IFN-γ production by CD8^+^ T cells cocultured with MDSCs derived from esophageal tumors models before and after treatment with GPR84 antagonist analyzed by ELISA. **e** Changes in tumor volumes (measured from days 10 to 28 in an in vivo imaging system) after cell implantation before and after treatment with GPR84 antagonist. Statistical graph depicting the fluorescence imaging assessment of tumor volumes. **f** Measurement of the xenografts from days 10 to 29 after cell implantation before and after treatment with GPR84 antagonist. **g** Measurement of lung metastasis before and after treatment with GPR84 antagonist using an in vivo imaging system. **h** Percentages of MDSCs and CD8^+^ T cells analyzed in the spleen of B16 tumor-bearing mice with the treatment of GPR84 antagonist using flow cytometry. **i** Percentages of IFN-γ, CD69, Ki67, CD28, and CD107a in CD8^+^ T cells from melanoma xenografts before and after treatment with GPR84 antagonist analyzed by flow cytometry. Proliferation (**j**) and IFN-γ production (**k**) by CD8^+^ T cells cocultured with MDSCs from melanoma xenografts analyzed by flow cytometry. Data are represented as mean ± SEM. *p < 0.05, **p < 0.01

Next, the GPR84 antagonist was further used to verify the effects on tumor microenvironment. GPR84 antagonist or dimethyl sulfoxide (DMSO) treatment was started on day 113 after 4-NQO challenge in orthotopic esophageal cancer. After two weeks of continuous treatment, normal drinking water was provided to the mice for one week, after which the spleen and tumor tissues were collected for analysis. The weight loss in GPR84 antagonist treatment group was significantly decreased (Fig. 5a). And the proportion of MDSCs decreased, whereas CD8^+^ T cell frequency increased with GPR84 blockade (Fig. 5b, c). Moreover, the inhibitory effects of MDSCs on CD8^+^ T cell proliferation and IFN−γ secretion were reversed (Fig. 5d). It was also found that treatment with GPR84 antagonist strangely inhibited melanoma growth (Fig. 5e, f) and decreased lung metastasis of malignant melanoma (Fig. 5g). It further decreased MDSCs and increased CD8^+^ T cell accumulation in the spleen (Fig. 5h). The activation and function signatures of CD8^+^ T cells (IFN-γ, CD69, Ki67, CD28, and CD107a) were also clearly upregulated in the GPR84 antagonist-treated group (Fig. 5i). Coincubation experiments showed that the immunosuppressive activity of MDSCs on the proliferation and IFN-γ secretion of CD8^+^ T cells were significantly reduced upon treatment with the GPR84 antagonist (Fig. 5j, k).

### GPR84 was induced by GM-CSF and G-CSF

**Fig. 6:**
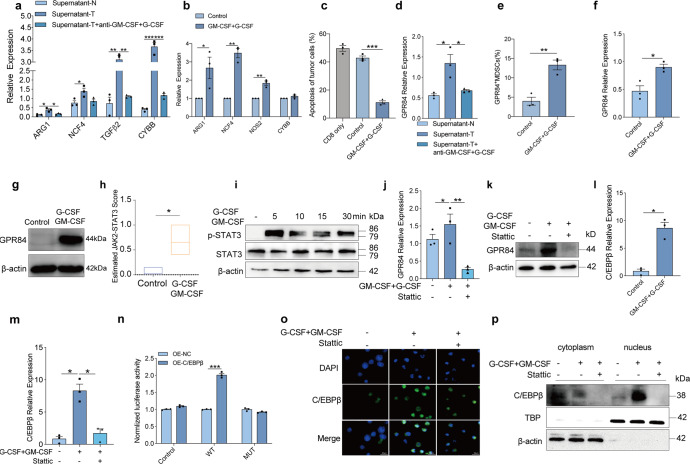
GPR84 is induced by GM-CSF and G-CSF. **a**, **d** Expression of immunosuppressive molecules (ARG1, NCF4, TGF-β2, and CYBB) in MDSCs isolated from PBMCs in the presence of culture medium of tumor tissue (supernatant-T) and normal tissue (supernatant-N) of esophageal cancer patients with or without antibody of GM-CSF and G-CSF analyzed using qPCR (*n* = 3 per group). Bone marrow cells derived from normal mice were cultured with or without GM-CSF and G-CSF recombinant protein for 3 days; then, the relative expression of immunosuppressive molecules (CYBB, NCF4, NOS2, and ARG1) (**b**) and cell apoptosis of B16-OVA cells cocultured with OT-1 CD8^+^ T cells (**c**) were examined using qPCR and flow cytometry, respectively. Percentages of GPR84^+^ MDSCs (**e**), mRNA (**f**) and protein (**g**) levels of GPR84 expression in bone marrow cells were analyzed by using flow cytometry and qPCR, respectively. **h** SSGSGA evaluations of estimated JAK2-STAT3 scores according to RNA-seq data, followed by *t*-tests for difference calculation. **i** Bone marrow cells were treated with or without GM-CSF and G-CSF at different times, with the expression of p-STAT3 and STAT3 assessed by western blot. The mRNA (**j**) and protein (**k**) expression of GPR84 was detected in the presence of GM-CSF and G-CSF with or without Stattic, the STAT3 inhibitor, using qPCR and western blot, respectively. **l** Expression of C/EBPβ in bone-marrow cells were investigated with or without GM-CSF and G-CSF treatment using qPCR. **m** Expression of GPR84 in the presence of GM-CSF and G-CSF with or without Stattic (STAT3 inhibitor) using qPCR. **n** 293 T cells were cotransfected with a wild-type (*WT*) or mutant (*MUT*) GPR84 promoter-luciferase reporter PGL3 and C/EBPβ overexpression plasmid for 24 h and were then assessed by luciferase assays. C/EBPβ expression in bone marrow cells in the presence of GM-CSF and G-CSF with or without Stattic treatment using immunofluorescence (**o**) and western blot assays (**p**). Data are represented as mean ± SEM. *p < 0.05, **p < 0.01

To identify the cytokines induced GPR84 expression in tumor microenvironment, the supernatants from ESCC tumor tissues were used. It was firstly found that supernatants from tumor significantly enhanced the expression of immunosuppressive molecules in myeloid cells (Fig. 6a). Granulocyte-macrophage colony-stimulating factor (GM-CSF) and granulocyte colony-stimulating factor (G-CSF) have been reported to induce MDSCs with an immunosuppressive phenotype.^20^ We also verified that MDSCs were obtained with a high level of immunosuppressive molecules in the presence of GM-CSF and G-CSF in vitro (Fig. 6b). In the coincubation system, MDSCs challenged with GM-CSF in combination with G-CSF inhibited the antitumor specific response of CD8^+^ T cells derived from OT-1 transgenic mice (Fig. 6c). With the neutralization of GM-CSF and G-CSF, we found that the expression of GPR84 and immunosuppressive molecules induced by esophageal carcinoma supernatant were both reversed (Fig. 6a, d). GM-CSF and G-CSF were also identified to enhance the expression of GPR84 in MDSCs derived from bone marrow cells of tumor-free mice (Fig. 6e–g). These findings indicate that GM-CSF and G-CSF promote the expression of GPR84 in MDSCs.

Next, a GPR84 antagonist was used to restrict the immunosuppressive function of MDSCs induced by GM-CSF and G-CSF. Firstly, MDSCs were purified by flow cytometry from esophageal cancer tissue and cultured with GM-CSF combined with G-CSF. The GPR84 antagonist addition downregulated the expression or secretion of immunosuppressive molecules (Supplementary Fig. 5a and b). In inducible MDSCs from bone marrow cells by GM-CSF combined with G-CSF, treated with GPR84 antagonist also significantly inhibited the expression and activity of immunosuppressive factors (Supplementary Fig. 5c and d).

Then, the molecular mechanism by which GM-CSF and G-CSF regulate GPR84 expression in MDSCs was determined by RNA-sequencing analysis. STAT3 can be activated by GM-CSF, which plays a crucial role in the expansion and suppressive activity of MDSCs.^21^ Consistent with this report, mRNA-sequencing data from GEO database showed that the Estimated JAK2-STAT3 score was elevated in G-CSF and GM-CSF treatment group, indicating a potential role of STAT3 in modulating GPR84 levels in MDSCs (Fig. 6h). To further explore whether GM-CSF and G-CSF might induce GPR84 expression through STAT3 activation, bone-marrow cells derived from tumor-free mice were treated with GM-CSF and G-CSF. As expected, STAT3 phosphorylation was activated (Fig. 6i). This activation was blocked by a STAT3 inhibitor (Fig. 6j, k). The transcription factors that may bind to the promoter region of GPR84 was predicted via the *PROMO* and *JASPAR* websites, and C/EBPβ was identified with the highest score (Supplementary Table 1). C/EBPβ has also been identified as an important transcription factor for MDSCs development induced by GM-CSF and G-CSF,^20^ so it was hypothesized that C/EBPβ mediates the induction of GPR84 by GM-CSF and G-CSF. GM-CSF and G-CSF were then verified to increase the expression of C/EBPβ, which was reversed by STAT3 inhibition (Fig. 6l, m). To investigate whether C/EBPβ physically binds to the promoter region of GPR84, a dual luciferase reporter assay was performed. And it was found that C/EBPβ overexpression promoted luciferase activity (Fig. 6n). In addition, the translocation of C/EBPβ into the nucleus was facilitated in the presence of GM-CSF and G-CSF, whereas inhibited by a STAT3 inhibitor (Fig. 6o, p). Taken together, these results indicate that GM-CSF and G-CSF promote the expression of GPR84 through STAT3- C/EBPβ activation.

### PD-L1 degradation was increased in GPR84-deficient MDSCs

**Fig. 7:**
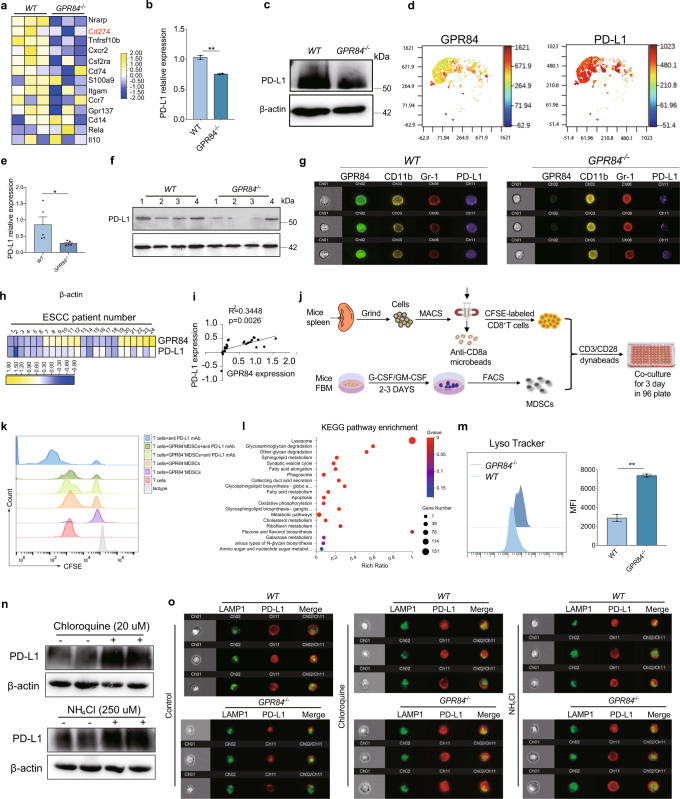
PD-L1 degradation increases in GPR84^−/−^ MDSCs. **a** Heat maps of RNA-seq data from tumor tissues were performed. Representative genes associated with MDSCs from each category are shown. **b**, **c** PD-L1 mRNA and protein expression in MDSCs induced by bone marrow cells isolated from *GPR84*^*−/−*^ and *WT* normal mice were measured by qPCR and western blot. **d** The Dimension reduction analysis of PD-L1in GPR84^+^ MDSCs induced by GM-CSF and G-CSF. **e**, **f** The mRNA and protein levels of PD-L1 expression in spleen MDSCs isolated from *GPR84*^*−/−*^ and *WT* tumor-bearing mice were measured by qPCR and western blot. **g** Imaging flow cytometry was used to examine PD-L1 expression in MDSCs. **h**, **i** Heatmap showing expression of CD33, PD-L1, and GPR84 in PBMCs derived from esophageal cancer patients. **j** The schematic diagram shows the co-incubation process of in vitro induced MDSCs and CD8^+^ T cells. **k** The proliferation of CD8^+^ T cells in different MDSCs coculture group and anti-PD-L1mAb treated group. **l** KEGG analysis of the RNA-seq data from *WT* and *GPR84*^*−/−*^ MDSCs derived from B16 tumor-bearing mice. **m** Fluorescence intensities of Lysotracker were measured in *GPR84*^*−/−*^ MDSCs induced by GM-CSF and G-CSF by flow cytometry. **n**, **o** Western blot and imaging flow cytometry were performed to investigate the effect of GPR84 on PD-L1 expression in the absence and presence of lysosomal inhibitors NH4Cl and chloroquine. Data are represented as mean ± SEM. *p < 0.05, **p < 0.01

For further exploring the mechanism underlying GPR84 promoting MDSCs suppression, whole-genome RNA sequencing was performed on tumor tissues derived from *WT* and *GPR84*^*−/−*^ LLC model. Deletion of GPR84 resulted in significant upregulation of 592 genes and downregulation of 9896 genes (Supplementary Fig. 6a). Enrichment analysis of MDSCs-associated genes revealed that a markedly decrease of CD274 expression in *GPR84*^*−/−*^ group (Fig. 7a). CD274, known as PD-L1, has been reported as the significant mechanism to inhibit CD8^+^ T cells. Inducible MDSCs by GM-CSF and G-CSF from bone-marrow cells were used to confirm that PD-L1 expression was reduced in *GPR84*^−/−^ MDSCs. (Fig. 7b, c and Supplementary Fig. 6b–d). Dimension reduction analysis also showed that PD-L1 was mainly expressed on GPR84^+^MDSCs induced by GM-CSF and G-CSF (Fig. 7d). MDSCs derived from *GPR84*^*−/−*^ LLC bearing mice also showed decreased PD-L1 expression (Fig. 7e, f, Supplementary Fig. 6e and f). Imaging flow cytometry was performed and verified the significant reduction of PD-L1 in *GPR84*^*−/−*^ MDSCs from LLC mice model (Fig. 7g). We also found that PD-L1 was positively correlated with GPR84 in MDSCs from the peripheral blood of esophageal cancer patients (Fig. 7h, i). With the treatment of GPR84 antagonist, the expression of PD-L1 was significantly reduced in MDSCs (Supplementary Fig. 6g). Fresh bone marrow cells were stimulated by GM-CSF + G-CSF for 3 days. Inducible MDSCs from bone marrow cells were incubated with CFSE labeled CD8^+^ T cells, and anti-PD-L1 mAb addition revealed that GPR84^+^ MDSCs inhibited CD8^+^ T cells proliferation dependent on PD-L1 (Fig. 7j, k).

To elucidate the mechanism of GPR84 promoting PD-L1 expression, we performed KEGG pathway analysis for the RNA-seq data of MDSCs derived from *GPR84*^*−/−*^ and *WT* LLC bearing mice. The lysosome-related genes were increased in *GPR84*^*−/−*^ MDSCs (Fig. 7l). We also found that the fluorescence intensity of LysoTracker was enhanced in *GPR84*^*−/−*^ MDSCs induced by GM-CSF and G-CSF (Fig. 7m). Coincubation of the selective lysosomal inhibitors chloroquine and NH_4_CL alleviated the downregulation of PD-L1 by GPR84 (Fig. 7n, o). These findings consistently demonstrated that the blockade of GPR84 promoted the lysosomal degradation of PD-L1.

### High risk of GPR84 signature inhibited anti-PD-1 efficiency

**Fig. 8:**
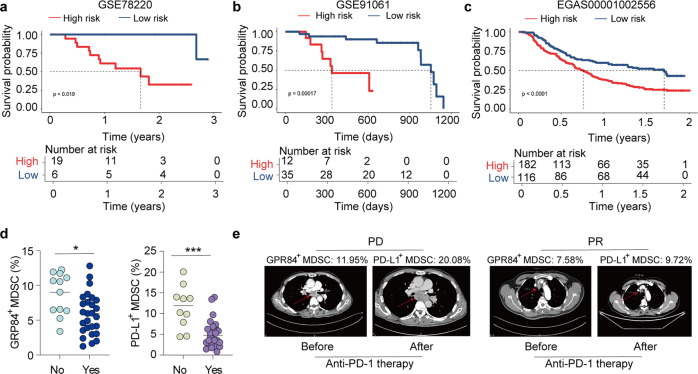
GPR84 high risk inhibits anti-PD-1 efficiency. **a**–**c** RNA-seq data from patients with cancer that received anti-PD-1 or PD-L1 treatment were downloaded from the GEO database or European Genome-Phenome Archive, respectively. According to the GPR84 signature, cancer patients were divided into high- and low-risk groups, then overall survival was compared using Kaplan–Meier analysis. **d** Peripheral blood from patients receiving combination chemotherapy and anti-PD-1 was collected to examine the expression of GPR84 and PD-L1 in MDSCs. **e** Imaging data from patients with and without reaction after anti-PD-1 treatment (PD: progressive diseases; PR: partial response). Data are represented as mean ± SEM. *p < 0.05, ***p < 0.001

Accumulation of MDSCs had been demonstrated with reducing anti-PD-1 efficacy. And the effect of GPR84 on MDSCs has been clarified in this study. So, next we will investigate the correlation between GPR84 expression and anti-PD-1 response in clinic. Patients with ESCC from TCGA were categorized into GPR84^high expression^ and GPR84^low expression^ groups. KEGG pathway enrichment analysis showed that the differential genes were enriched in the pathway of the cytokine-receptor, PD-L1, and PD-1 (Supplementary Fig. 7a). To further clarify the clinical effect of anti-PD-1 therapy on the expression and functional characteristics of GPR84, we compared the RNA-seq data from GPR84^+^ and GPR84^-^ MDSCs of patients with ESCC (Supplementary Fig. 7b). The top ten differentially expressed genes are shown in Supplementary Fig. 7c. Then, the top ten genes expression were defined as the risk score of GPR84. Kaplan–Meier survival analysis revealed that anti-PD-1-treated patients who were obtained with a low GPR84 risk score had good survival (Fig. 8a–c). Peripheral blood of patients receiving chemotherapy and anti-PD-1 combination was collected to determine whether the expression of GPR84 and PD-L1 in MDSCs effects on the response to anti-PD-1 treatment (Supplementary Table 2). The patients with high levels of both GPR84^+^ MDSCs and PD-L1^+^ MDSCs had poor response to anti-PD-1 treatment (Fig. 8d). The imaging data from patients with and without response after anti-PD-1 treatment are shown in Fig. 8e. Therefore, evaluation of the clinical data of patients with malignant tumors demonstrated that the GPR84 signature was positively correlated with resistance to anti-PD-1 therapy.

### GPR84 antagonism combined with anti-PD-1 antibody treatment enhances antitumor immunity

**Fig. 9:**
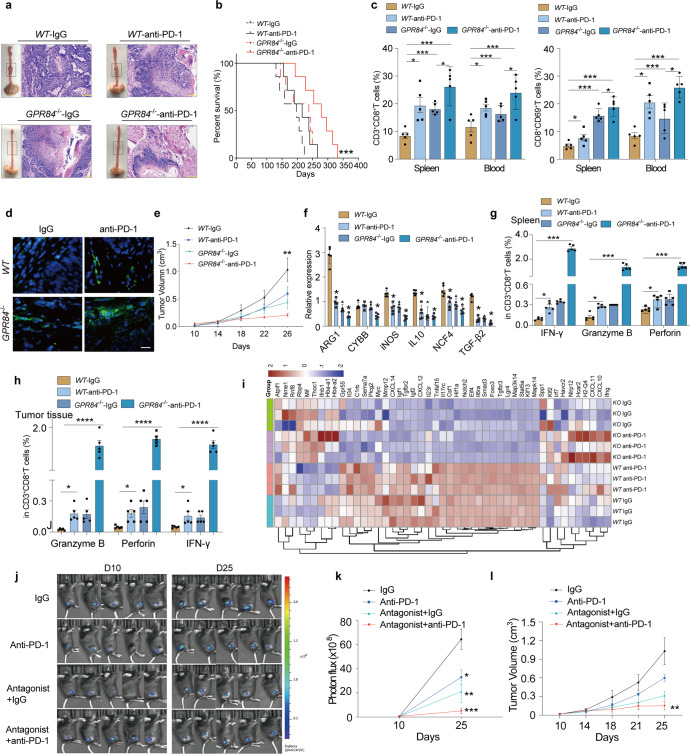
GPR84 antagonism combined with anti-PD-1 therapy enhances antitumor immunity. **a** Gross and microscopic specimens of esophageal tumors produced in response to 4-NQO stimulation in *WT* and *GPR84*^*−/−*^ mice administered anti-PD-1 or IgG. **b** Overall survival of 4-NQO-challenged *WT* and *GPR84*^*−/−*^ mice administered anti-PD-1 or IgG, analyzed by the Kaplan–Meier method. **c** Percentages of CD3^+^CD8^+^ T cells and CD8^+^CD69^+^ T cells in the peripheral blood and spleen of 4-NQO-challenged *WT* and *GPR84*^*−/−*^ mice treated with anti-PD-1 or IgG were analyzed by flow cytometry. **d** Immunofluorescence analysis of CD8 (green) expression in esophageal cancer tissues from 4-NQO-challenged *WT* and *GPR84*^*−/−*^ mice treated with anti-PD-1 or IgG; DAPI (blue), CD8 (green). **e** Tumor volumes in *WT* and *GPR84*^*−/−*^ mice administered anti-PD-1 or IgG measured from days 10 to 26 after LLC-cell injection. Relative expression of ARG1, CYBB, iNOS, IL-10, NCF4, and TGF-β2 in purified MDSCs (**f**) and percentages of IFN-γ, granzyme B, and perforin in CD3^+^CD8^+^ T cells from spleen (**g**) and tumor tissues (**h**) of LLC-cell-injected *WT* and *GPR84*^*−/−*^ mice administered anti-PD-1 or IgG analyzed by qPCR and flow cytometry, respectively. **i** Heat map depicting the expression of T cell activation and function-related molecules in *WT* and *GPR84*^*−/−*^ mice administered anti-PD-1 or IgG treatment. **j** Tumor volumes measured on days 10 and 25 after LLC-cell-luciferase implantation before and after treatment with anti-PD-1 and GPR84 antagonist using an in vivo imaging system. **k** Statistical graph depicting the tumor volumes as a function of fluorescence intensity. **l** Measurement of the xenografts from days 10 to 25 after cell implantation before and after administration of anti-PD-1 and GPR84 antagonist. Data are represented as mean ± SEM. *p < 0.05, **p < 0.01, ***p < 0.001, ****p < 0.0001

Using *GPR84*^*−/−*^ mice or GPR84 antagonist, we found that tumor progression was inhibited dependent on the prevention of MDSCs immunosuppressive activity. We further evaluated whether GPR84 antagonism could be combined with anti-PD-1 treatment to improve antitumor immunotherapy. Beginning on day 113 of tumor induction with 4-NQO, the *WT,* and *GPR84*^*−/−*^ groups were treated with anti-PD-1 or IgG every three days (Supplementary Fig. 8a). Anti-PD-1 treatment notably reduced tumor growth (Fig. 9a) and prolonged the survival of *GPR84*^*−/−*^ mice (Fig. 9b). It also showed that accumulation of total CD8^+^ T cells and activated subpopulations was higher in *GPR84*^*−/−*^ mice treated with anti-PD-1 than in *WT* mice (Fig. 9c). Immunofluorescence confirmed high infiltration of CD8^+^ T cells in the *GPR84*^*−/−*^ group treated with anti-PD-1 (Fig. 9d).

Subcutaneous tumor model of LLC was constructed in *WT* and *GPR84*^*−/−*^ mice (Fig. S8b). In *GPR84*^*−/−*^ mice treated with anti-PD-1, tumor volume was significantly reduced (Fig. 9e), and immunosuppression-related genes in MDSCs from tumor tissues were significantly downregulated (Fig. 9f). More interestingly, the expression of IFN-γ, Granzyme B, and Perforin on CD8^+^ T cells in GPR84^−/−^ mice treated with anti-PD-1 increased (Fig. 9g, h). Similarly, RNA-seq data showed that the expression of T cell activation- and function-related molecules was significantly increased in *GPR84*^*−/−*^ mice treated with anti-PD-1 (Fig. 9i). To investigate the efficacy of combination therapy with *GPR84* blockade and anti-PD-1, subcutaneous tumor models were constructed using LLC luciferase cells (Supplementary Fig. 8c). Tumor fluorescence intensity measurements and growth curves demonstrated that the combination therapy inhibited tumor growth (Fig. 9j–l). The accumulation and activation of CD8^+^ T cells were significantly increased (Supplementary Fig. 8d and e) in mice treated with combination therapy. These results indicate that the deficiency or blockade of GPR84 restored the response of anti-PD-1 treatments, which developed a new therapeutic strategy for tumor immunotherapy.

## Discussion

Immunosuppression associated with MDSCs plays an important role in tumor progression.^1,22^ In this study, we showed that GPR84 facilitates the suppressive phenotype and function of MDSCs. GPR84 was prominently expressed on MDSCs, which promoted the accumulation and immunosuppressive function of MDSCs, interfering with the antitumor immunity of T cells and leading to a reduction in the effectiveness of anti-PD-1. Furthermore, we found that G-CSF plus GM-CSF, induced the expression of GPR84 on MDSCs via the STAT3/ C/EBPβ signaling pathway. Mechanistically, blocking GPR84 reduced PD-L1 expression depending on lysosome degradation. Similar modulation by lysosomes on PD-L1 expression has also been reported by Wang et al.^23^ In addition, we found that GPR84^+^MDSCs worked as a negative indicator of anti PD-1 therapy in ESCC and NSCLC patients. Finally, we found that applying a GPR84 antagonist significantly enhanced the efficiency of anti-PD-1 therapy in vivo.

MCFAs are the only natural ligands for GPR84, and endogenous ligands remain unknown.^19^ The only reported study on GPR84 related to tumors focused on its effects on stem-cell proliferation in leukemia.^18^ GPR84 agonists, such as 6-n-octylaminouracil, can induce the activation of intracellular signaling pathways, including ERK, PI3K, and Akt-mediated pathways in vitro, leading to the release of IL-6, IL-10, VEGF, and MIP-2.^16,24^ Although none of these studies have evaluated the effect of GPR84 on the TME, cytokines induced by GPR84 have been reported to promote tumor progression in multiple solid tumors. Herein, we observed that loss of GPR84 restrained tumor growth, consistent with a role for GPR84 in solid tumor progression.

Numerous studies have demonstrated a major role for local immunosuppression in inhibiting robust antitumor responses, and MDSCs are key regulators of immunosuppressive networks.^1^ GPR84 was predominantly expressed on MDSCs. In subsequent experiments, GPR84 selective antagonists interfered with the accumulation and immunosuppression of MDSCs. The GPR84 selective antagonist restored the proliferation ability and antitumor immunity of CD8^+^ T cells, thereby delaying tumor progression in mouse models. This is the first report to clarify the distribution patterns of GPR84 in solid tumors and reveal its importance in the MDSC-mediated immunosuppressive microenvironment.

MDSCs promote resistance to anti-PD-1 therapy by inhibiting the proliferation of T cells in solid tumors.^25^ The above results indicated that GPR84, which is prominently expressed on MDSCs, could be targeted therapeutically in vivo. In this study, combining anti-PD-1 therapy with inhibition or deletion of GPR84 significantly increased antitumor immunity. This was similar to other methods aimed at limiting MDSCs activity to increase CD8^+^ T cell responses. Moreover, T cell proliferation- and activation-related genes were significantly upregulated in the GPR84-deficient and combination treatment groups. Although this study was performed only in mouse models, another GPR84 selective antagonist, PBI-4050, has been used in phase II clinical trials to treat inflammatory bowel disease. This suggests the safety of GPR84-targeted therapy in clinical applications. Therefore, targeting GPR84 is a promising combination therapy approach with anti-PD-1 to enhance the antitumor immune response.

In summary, MDSCs highly expressing GPR84 are critical for the suppression of antitumor immunity via inhibiting PD-L1 degradation in lysosome. GPR84 blockade inhibits tumor progression by remodeling the immunosuppressive microenvironment. Therefore, combination therapy targeting GPR84 and anti-PD-1 is a potential therapeutic strategy for solid tumors.

## Materials and methods

### RNA sequences

Genbank

### Sequencing data

GSE71706, GSE21927, GSE145370, GSE78220, GSE91061, EGAS00001002556.

The data from TCGA generated during this study are available at http://www.cbioportal.org.

### Online analysis

*PROMO* (http://alggen.lsi.upc.es/)

*JASPAR* (http://jaspar.genereg.net/)

The detailed reagent materials were displayed in the KEY RESOURCES TABLE in Supplementary data.

### Study approval

All mouse experiments were approved by the Henan Key Laboratory for Pharmacology of Liver Diseases (approval 00018170). All participants provided written informed consent. This study was approved by the Ethics Committee of the First Affiliated Hospital of Zhengzhou University (approval 2019-KY-256).

### Animal models

Animals were maintained under pathogen-free conditions and provided care in accordance with the International Association for Assessment and Accreditation of Laboratory Animal Care policies and certification. Four-to-six-week-old female C57BL/6J mice were purchased from the Beijing Charles River Company (Beijing, China). C57BL/6J–GPR84/BRL mice were provided by BRL^+^ Medicine (Shanghai, China). OT-1 mice were kindly provided by the Bo Huang lab of the Institute of Basic Medicine, Chinese Academy of Medical Sciences.

The mouse model of orthotopic esophageal cancer was generated by challenge with 4-NQO added to drinking water.^26^ A 5 mg/mL stock solution was prepared by dissolving 4-NQO in 5 mL of propylene glycol. Next, 3 mL of the stock solution was mixed with 147 mL of autoclaved water to a final concentration of 100 μg/mL) for drinking water. Drinking water was changed 1–2 times per week. The control group was provided 147 mL of drinking water mixed with 3 mL of propylene glycol. To construct mouse models of lung cancer and malignant melanoma for further study, LLC (5 × 10^5^) and B16 (2 × 10^5^) cells were subcutaneously injected. Once the tumor volume reached 50 mm^3^, the mice were randomly divided into two groups. A GPR84 antagonist was administered through drinking water at a dose of 50 mg kg^−1^ day^−1^. The control group was administered the same concentration of the solvent. Anti-PD-1 monoclonal antibody (BE0146, clone RMP1-14; Bio-XCell) or IgG isotype control (BE0089, clone 2A3; Bio-XCell) was administered at 10 mg/kg intraperitoneally once every three days, for a total of four injections.^27^ Esophageal cancer was evaluated based on the amount of food consumed, water intake, or body weight of the mice. Mice were euthanized on days 0, 113, and 162; tissue sections stained with hematoxylin and eosin were examined to assess tumor development. On day 135 after 4-NQO treatment, *WT* and GPR84^*−/−*^ mouse models of esophageal cancer were sacrificed for further experiments. Lung cancer and malignant melanoma were assessed by evaluating tumor growth. To observe the effect of GPR84 deletion on MDSCs and CD8^+^ T cells in LLC and B16F0 models, tumor-bearing mice were sacrificed on the 20th day after LLC injection and the 22nd day after B16F0 challenged, respectively.

### Clinical samples

Peripheral blood (10 mL) was collected from healthy donors aged 40–70 years and from patients with esophageal squamous cell carcinoma or lung adenocarcinoma. All patients were diagnosed via pathological examination. Mononuclear cell suspensions (peripheral blood mononuclear cells) were obtained by density-gradient centrifugation in lymphocyte separation solution. Labeled antibodies and MDSCs or CD8^+^ T cells were separately detected and purified according to experimental requirements.

Tumor and paired paracancerous tissues from patients with esophageal squamous cell carcinoma and lung adenocarcinoma cancer, confirmed by pathological diagnosis, were collected under sterile conditions, placed in RPMI 1640 medium containing 10% FBS, 10% penicillin, and 10% streptomycin, and immediately sent to the laboratory. All analyzed samples were confirmed by pathology. The Miltenyi CliniMACS (Cologne, Germany) system was used to obtain single-cell suspensions from tumor tissues, followed by flow cytometric detection or sorting of target cells. Samples used for immunohistochemistry and immunofluorescence were provided by the Department of Pathology, First Affiliated Hospital of Zhengzhou University, and the Henan Key Laboratory of Esophageal Cancer (Zhengzhou, China).

### Cell lines and culture conditions

All cell lines were tested to confirm that they were free of mycoplasma or other pathogens. The murine lung-cancer cell lines LLC, B16, and B16-OVA were purchased from the American Type Culture Collection (Manassas, VA, USA). Cells were cultured at 37 °C in 5% CO_2_ in Dulbecco’s modified Eagle medium supplemented with 10% FBS, penicillin, and streptomycin.

### Isolation of tumor-infiltrating cells

Tumor tissues from mice and surgery patients were washed with PBS to remove impurities, cut into ~1-cm^3^ pieces, and placed in a gentleMACS™ C tube (Miltenyi). Next, 4.7 mL of serum-free RPMI 1640 medium was added to the tissues, followed by mechanical dissociation using a tri-enzyme solution (325 μL). The solution was filtered, and cell pellets were collected for subsequent experiments.

### Isolation of immune cells

Mice in which 4-NQO had induced the formation of esophageal tumors were selected; these tumors were used to prepare single-cell suspensions, which were divided into three groups. Group 1 was labeled with CD3, CD8, and CD49b; group 2 was labeled with CD3, B220, and CD11c; and group 3 was stained with Gr1, CD11b, and F4/80. Single-cell suspensions from patients with esophageal cancer were prepared to isolate CD11b^+^ cells using model-based analysis of chromatin immunoprecipitation sequencing, as CD11b^+^ cells express CD33, 7-AAD, HLA-DR, CD11b, and GPR84. After incubation with antibodies at 4 °C for 20 min, cells were washed with PBS to remove unbound antibodies. Cells were resuspended in 500 μL of RPMI 1640 medium containing 10% FBS, then aliquoted into a sterile flow-type loading tube by passing through a 200-mesh strainer. For sorting, gating parameters were selected based on different target cells. After fixing the sorting position, the analysis was stopped, and sorting was initiated. Cells of interest were collected in a flow tube containing serum-free RPMI 1640 medium.

### Flow cytometry

Single-cell suspensions prepared from peripheral blood, tumor tissue, or mouse spleen tissue were counted and 1 × 10^6^ cells were used for staining. After vortexing, labeling reactions were incubated at 4 °C in the dark for 15 min, followed by washing with 1 mL PBS and data acquisition with fluorescence-activated cell sorting (BD FACSCanto II). Export FCS files and FlowJo 10.4 software were used for data analysis.

### MDSCs induction

Bone-marrow cells were obtained from 6-week-old C57BL/6 mice and divided into control, GM-CSF combined with IL-6, and GM-CSF combined with G-CSF treatment groups. The working concentration of cytokines was 50 ng/mL. After culturing for 72 h, cells were harvested and labeled for flow cytometry with antibodies against Gr1, Ly6G, Ly6C, 7-AAD, and GPR84 to determine MDSCs proportions and GPR84 expression. MDSCs from different groups were collected to analyze functional molecules, such as CYBB, NCF4, NOS2, and ARG1, by real-time quantitative PCR.

### Sorting MDSCs and CD8^+^ T cells

After obtaining single-cell suspensions, red blood cells were lysed and an isolation kit (Miltenyi) was used to purify MDSCs or CD8^+^ T cells from clinical samples and tumors from the mouse models. The sorting efficiency of MDSCs or CD8^+^ T cells by flow cytometry was >90%.

### Inhibitory effect of MDSCs on CD8^+^ T cells

The CFSE fluorescent probe labeling method was used to detect the proliferation of CD8^+^ T cells collected from clinical samples or tumor-bearing mice. Cells were mixed with CFSE (5 μmol/L) for labeling. Labeled cells were incubated with MDSCs from different sources (MDSCs:CD8 = 4:1), with individual CD8^+^ T cells used as controls. CD3/CD28 Dynabeads were added to stimulate CD8^+^ T cell proliferation. After 48 h, the fluorescence of fluorescein isothiocyanate (FITC) was analyzed by flow cytometry to measure CD8^+^ T cell proliferation. Supernatants were collected for IFN-γ detection by ELISA to measure differences in CD8^+^ T cell function between the control and coculture groups.

### In vitro cytotoxicity assay

CD8^+^ T cells were obtained from the spleens of 6-week-old OT-1 transgenic mice using magnetic bead sorting. B16F0-OVA cells labeled with CFSE were incubated with CD8^+^ T cells (5:1) and MDSCs from different sources were added (MDSCs:CD8 = 4:1) to interfere with the interaction. After incubation at 37 °C for 8 h, percentages of apoptotic tumor cells were measured by flow cytometry. Propidium (PI) was added before detected by flow cytometry. The double positive cells with PI and CFSE were apoptotic tumor cells.

### Quantitative RT-PCR

Total RNA from MDSCs, T cells, B cells, NK cells, and macrophages was isolated using TRIzol Reagent (Invitrogen Life Technologies, Carlsbad, CA, USA). Reverse transcription was performed using a PrimeScript RT reagent kit, following the manufacturer’s instructions (Takara, Shiga, Japan). Gene expression was detected using SYBR Green qPCR Master Mix (Takara) on an MX3005P qPCR system (Agilent Technologies, Santa Clara, CA, USA). Relative mRNA expression was calculated using the 2^−ΔΔCt^ method.

### Immunohistochemistry and immunofluorescence

Deparaffinized sections were subjected to antigen retrieval, followed by endogenous oxidase inactivation. After blocking in PBS containing 2% bovine serum albumin, primary antibodies for GPR84 or control anti-rabbit IgG were added and incubated overnight at 4 °C. Samples were then stained with anti-rabbit antibody labeled with horseradish peroxidase. Slides were analyzed by Pannoramic Scanner, and H-score was used to calculate expression levels for the statistical analysis of overall survival.

Dewaxing, antigen retrieval, and blocking for immunofluorescence detection were similar to those used for immunohistochemistry. Primary antibodies were added and incubated overnight at 4 °C. After washing with PBS, secondary antibodies were added for 1 h in the dark. Nuclei were counterstained with DAPI and slides were analyzed by fluorescence microscopy and Case Reviewer software.

### Hematoxylin-eosin staining

Sections (3 µm) from normal esophageal tissues or 4-NQO challenged esophageal cancer tissue were placed in a 60 °C oven for 1.5 h. After dewaxing, hematoxylin staining was performed at room temperature for 10 min, washed with running water, and then placed in 0.7% hydrochloric acid and ethanol for 10 s. Eosin staining was performed at room temperature; then, sections were passed through increasing concentrations of ethanol and xylene for rehydration.

### Dual-luciferase reporter assay

HEK 293T cells were transfected with the Renilla luciferase plasmid pRL-SV40, firefly luciferase plasmid pGL3-WT or MUT, and C/EBPβ overexpression plasmid in a 1:1:1 ratio for 24 h according to the manufacturer’s instructions. Cell lysates were analyzed using the Dual Luciferase Reporter Assay Kit (E2910, Promega) on a microplate reader. Firefly luciferase activity was normalized to Renilla luciferase activity for each sample.

### ROS detection

MDSCs purified from healthy volunteers and patients with esophageal carcinoma were resuspended in 1 mL serum-free RPMI 1640 medium mixed with the fluorescent probe 2,7-dichlorofluorescin diacetate (DCFH-DA) (10 μmol/L) and incubated at 37 °C for 20 min, with inversion and mixing every 5 min. Cells were washed three times with serum-free RPMI 1640 medium. The presence of ROS was evaluated based on FITC fluorescence using flow cytometry.

### ARG1 activity detection

Arginase is a manganese-containing enzyme that catalyzes the conversion of arginine to urea and ornithine. MDSCs from different sources were collected, cell lysates were added, and arginase activity was detected simultaneously using a urea standard working solution. Substrate buffer was added, incubated for 2 h, and urea reagent was used to stop the arginase reaction. The absorbance of each well at a wavelength of 430 nm was measured using a multi-functional microplate reader and calculated using the formula:

$${\rm{Activity}} = \frac{{\left( {A_{430}} \right)_{\rm{sample}} \,-\, \left( {A_{430}} \right)_{\rm{blank}}}}{{\left( {A_{430}} \right)_{\rm{standard}} \,-\, \left( {A_{430}} \right)_{\rm{water}}}} \times \frac{{\left( {1mM \times 50 \times 10^3} \right)}}{{\left( {V \times T} \right)}}$$

### Lysosomal inhibition

MDSCs (2 × 10^6^/well) were treated with G-SCF and GM-SCF for 48 h. Then, the selective lysosomal inhibitor NH4Cl (250 μM) or chloroquine (20 μM) was added, followed by incubation for 24 h at 37 °C with 5% CO_2_. After incubation, the cells were washed three times with FACS buffer (0.5% FBS in 1 × PBS). The primary antibody, LAMP1 (ab208943, Abcam, 1:500) was added and incubated for 30 min on ice in the dark. Cells were washed three times by centrifugation at 1500 rpm for 5 min and resuspended in FACS buffer. Fluorochrome-labeled secondary antibodies Alexa Fluor®R555 (FcMACS, 1:1000) and streaming antibodies (CD11b, Gr1, and PD-L1) were added and incubated for 30 min on ice in the dark. Cells were then washed three times by pelleting at 1500 rpm for 5 min and resuspended in FACS buffer. Cells were analyzed using a FlowSight Imaging flow cytometer (Amnis). Data were analyzed using IDEAS software (Amnis).

### RNA sequencing

Fresh GPR84^+^ and GPR84^−^ MDSCs were sorted from esophageal cancer tissue and RNA was extracted using a Qiagen Allprep kit (Hilden, Germany). Similarly, LLC tumors from WT or GPR84^−/−^ mice administered anti-PD-1 or isotype mAb were collected and RNA was extracted using the same method. Approximately 1 µg of RNA was used by Novogene for library construction and sequencing. Messenger RNA profiles were generated based on single-read deep sequencing in triplicate using an Illumina HiSeq2000 (San Diego, CA, USA).

### mRNA sequencing data analysis

Sequence files from Illumina HiSeq that passed quality filters were aligned against the mouse transcriptome using Bowtie2 aligner4. Gene-level count summaries were analyzed for significant changes using DESeq. Individual *p*-values were adjusted for multiple testing by calculating Storey’s *q*-values using the fdrtool trimmer. For each gene, the q-value had the smallest false discovery rate at which the gene was significant. Biological processes were analyzed according to the guidelines provided by the Gene Ontology Consortium, in which each gene ontology term defines a set of genes. The entire list of genes, sorted based on *q*-values in ascending order, was subjected to a non-parametric variant of GSEA, in which the parametric Kolmogorov-Smirnov *p*-value was replaced with the exact rank-order *p*-value. Heatmaps depicting the expression levels were generated using an in-house hierarchical clustering software that implements Ward clustering. The colors qualitatively correspond to fold changes in expression. The mRNA-sequencing data reported in this study have been deposited in Genome Sequence Archive (Genomics, Proteomics & Bioinformatics 2021) in the National Genomics Data Center (Nucleic Acids Res 2022), China National Center for Bioinformation / Beijing Institute of Genomics, Chinese Academy of Sciences (GSA-Human: HRA003874) that are publicly accessible at https://ngdc.cncb.ac.cn/gsa-human. The project number is PRJCA014498. And in this project, the data from LLC mice has been submitted in OMIX (https://ngdc.cncb.ac.cn/omix/submitList) as OMIX002872.

### TCGA database analysis

The mRNA sequencing data from patients with esophageal squamous cell carcinoma and lung adenocarcinoma carcinoma were downloaded from TCGA. Eight molecules related to the MDSCs phenotype or function (such as CD33) were selected for correlation analysis with GPR84. The results are displayed in a heat map generated using the corrplot package in R. Patients were divided into GPR84^high^ and GPR84^low^ groups based on GPR84 mRNA expression levels. Kaplan–Meier analysis was performed to calculate the difference in overall survival between GPR84^high^ and GPR84^low^ patients.

### Single-cell RNA sequencing data analysis

The single-cell RNA sequencing data of ESCC were downloaded from GEO database(GSE145370). we processed the unique molecular identifier (UMI) count matrix using the R package Seurat (version 3,2,2). As the quality control(QC), we first excluded genes detected less than ten cells and cells that have <200 nonzero count genes. We next filtered out the cells if their proportions of mitochondrial gene expression were larger than 10%. We used canonical correlation analysis (CCA) to aggregate each sample, respectively. After QC, 102611 cells and 18570 genes were included in downstream analysis. For clustering, we first run PCA and selected top 20 PCA to find clusters with resolution of 0.5, and then we performed run T‐distributed Stochastic Neighbor Embedding (tSNE) and the clusters were visualized by DimPlot in Seurat. The visualization of GPR84, ITGAM were used FeaturePlot in Seurat. All analysis were conducted by R(version 3.6.3).

### Statistical analyses

Statistical analyses were performed using GraphPad Prism 7 software (GraphPad, Inc., La Jolla, CA, USA) or R project. The Kaplan–Meier method was used to evaluate the effect of GPR84 on the overall survival of patients with esophageal cancer or lung adenocarcinoma cancer. Paired or unpaired *t-*tests were used to analyze the differences in GPR84 or other immunosuppressive molecule expression in different MDSCs. One- or two-way ANOVAs were performed to compare multiple conditions. The normality of all samples was tested using the Shapiro-Wilk test. Spearman correlation analysis was used to evaluate the correlation between GPR84 expression and MDSCs functional molecule expression.

## Supplementary information

Supplementary Materials

## Data Availability

The authors confirm that the data supporting the findings of this study are available within the article.
