# Supplementary material for: Overcoming resistance to immunotherapy by targeting GPR84 in myeloid-derived suppressor cells
Source: Signal Transduct Target Ther. 2023 Apr 28;8:164. doi: 10.1038/s41392-023-01388-6 (PMC10140025; doi:10.1038/s41392-023-01388-6)
Supplement: Supplementary file 1 — Supplementary Materials [file 41392_2023_1388_MOESM1_ESM.docx]

Supplementary Materials for

**Overcoming resistance to immunotherapy by targeting GPR84 in myeloid-derived suppressor cells**

Guohui Qin^#1^, Shasha Liu^#1^, Jinyan Liu^#1^, Hongwei Hu^1^, Li Yang^1^, Qitai Zhao^1^, Congcong Li^1^, Bin Zhang^3^, Yi Zhang^1, 2, 4, 5^**^*^**

^1^Biotherapy Center and Cancer Center, The First Affiliated Hospital of Zhengzhou University, Zhengzhou, Henan, 450052, China; ^2^School of Life Sciences, Zhengzhou University, Zhengzhou, Henan, 450001, China; ^3^Department of Medicine-Division of Hematology/Oncology, Feinberg School of Medicine Northwestern University, Chicago, IL 60611, USA; ^4^Henan Key Laboratory for Tumor Immunology and Biotherapy, Zhengzhou, Henan, 450052, China; ^5^State Key Laboratory of Esophageal Cancer Prevention & Treatment, Zhengzhou University, Zhengzhou, Henan, 450052, China

***Corresponding Author:**

Yi Zhang, MD, PhD

Biotherapy Center, The First Affiliated Hospital of Zhengzhou University; Zhengzhou 450052, China; Tel: +86 037166295320; E-mail: [yizhang@zzu.edu.cn](mailto:yizhang@zzu.edu.cn)

**This PDF file includes:**

Materials and Methods

Supplementary Text

Figures. S1 to S8

Tables S1 to S2

**Materials and Methods**

**KEY RESOURCES TABLE**

| REAGENT or RESOURCE | SOURCE | IDENTIFIER |
| --- | --- | --- |
| Antibodies |  |  |
| PE anti-mouse CD3 Antibody | BioLegend | Cat #100206;RRID:AB_312663 |
| APC anti-mouse CD8a Antibody | BioLegend | Cat #100712;RRID:AB_312751 |
| FITC anti-mouse CD19 Antibody | BioLegend | Cat #152404;RRID:AB_2629813 |
| PE/Cyanine7 anti-mouse CD49b Antibody | BioLegend | Cat #108921;RRID:AB_2561459 |
| PE anti-mouse Ly-6G/Ly-6C (Gr-1) Antibody | BioLegend | Cat #108408;RRID:AB_313373 |
| APC anti-mouse/human CD11b Antibody | BioLegend | Cat #101212;RRID:AB_312795 |
| APC/Cyanine7 anti-mouse F4/80 Antibody | BioLegend | Cat #123118;RRID:AB_893477 |
| FITC anti-mouse CD11c Antibody | BioLegend | Cat #117306;RRID:AB_313775 |
| APC/Cyanine7 anti-human CD14 Antibody | BioLegend | Cat #367108;RRID:AB_2566710 |
| FITC anti-human CD33 Antibody | BioLegend | Cat #303304;RRID:AB_314344 |
| PE/Cyanine7 anti-human HLA-DR Antibody | BioLegend | Cat #307616;RRID:AB_493588 |
| APC anti-human CD3 Antibody | BioLegend | Cat #300312;RRID:AB_314048 |
| FITC anti-human CD8a Antibody | BioLegend | Cat #300906;RRID:AB_314110 |
| PE anti-human CD4 Antibody | BioLegend | Cat #357404;RRID:AB_2562036 |
| PE anti-human CD56 Antibody | BioLegend | Cat #355503;RRID:AB_2561552 |
| APC anti-human CD19 Antibody | BioLegend | Cat #392503;RRID:AB_2728415 |
| APC Annexin V | BioLegend | Cat #640920 |
| PE/Cyanine7 anti-mouse Ly-6G Antibody | BioLegend | Cat #127618;RRID:AB_1877261 |
| APC/Cyanine7 anti-mouse Ly-6C Antibody | BioLegend | Cat #128026;RRID:AB_10640120 |
| FITC anti-mouse/human CD11b Antibody | BioLegend | Cat #101206;RRID:AB_312789 |
| CD11B Antibody | Proteintech | Cat # 21851-1-AP |
| Rabbit Anti-GPR84 antibody | Bioss | bs-13507R |
| Anti-GPR84/FITC | Bioss | bs-13507R-FITC |
| Anti-GPR84/PE | Bioss | bs-13507R-PE |
| CD33 Antibody | ZSGB-bio | TA336936 |
| Anti-CXCL1 antibody | R&D | Cat # MAB453-SP |
| Anti-IL-6 antibody | R&D | Cat # MAB206-SP |
| Anti-Rabbit IgG Fc antibody | Abcam | Cat # ab190492 |
| β-Actin (8H10D10) Mouse mAb | Cell Signaling Technology | Cat # 3700 |
| Phospho-NF-κB p65 Rabbit mAb | Cell Signaling Technology | Cat #3033 |
| Phospho-mTOR Rabbit mAb | Cell Signaling Technology | Cat #5536 |
| Phospho-Stat3 (Tyr705) (D3A7) XP® Rabbit mAb | Cell Signaling Technology | Cat #9145 |
| Phospho-p44/42 MAPK (Erk1/2) (Thr202/Tyr204) (D13.14.4E) XP® Rabbit mAb | Cell Signaling Technology | Cat #4370 |
| Phospho-p38 MAPK (Thr180/Tyr182) (D3F9) XP® Rabbit mAb #4511 | Cell Signaling Technology | Cat #4511 |
| Anti-iNOS | Abcam | ab178945 |
| Anti-Arginase1 | Abcam | ab203490 |
| Biological Samples |  |  |
| Human cancer tissues and paired paracancerous tissues | the Department of Pathology of the First Affiliated Hospital of Zhengzhou University and Henan Key Laboratory of Esophageal Cancer | Ethical Approval Number: Research-2010-LW-1215 |
| Chemicals, Peptides, and Recombinant Proteins |  |  |
| GPR84 antagonist 8 | MedChemExpress | CAS NO.: 1445846-30-9 |
| 4% paraformaldehyde | Solarbio | P1110 |
| Hematoxylin | Solarbio | G1080 |
| Eosin Y solution (Water Soluble) | Solarbio | G1100 |
| Propidium Iodide | Solarbio | C0080 |
| 4-NQO | Sigma | Lot#WXBD1945V |
| BioTracker 488 Green CSFE Cell Proliferation Kit | Sigma | SCT110 |
| Recombinant Human IL-2 | Peprotech | Cat # 200-02 |
| Dynabeads Human T-Activator CD3/CD28 | Thermo Fisher Scientific | 11132D |
| Recombinant Murine GM-CSF | Peprotech | Cat # 315-03 |
| Recombinant Murine M-CSF | Peprotech | Cat # 315-02 |
| Recombinant Murine IL-6 | Peprotech | Cat # 216-16 |
| InVivoMAb anti-mouse PD-1 (CD279) | Bio X Cel | Cat # BE0033-2 |
| InVivoMAb mouse IgG2b isotype control, unknown specificity | Bio X Cel | Cat # BE0086 |
| Critical Commercial Assays |  |  |
| ROS detection kit | Beyotime | S0033M |
| Mouse IFN-gamma ELISAPRO kit | MabTech | 3321-1HP-2 |
| Human IFN-gamma ELISAPRO kit | MabTech | 3420-1HP-2 |
| PrimeScript 1st strand cDNA Synthesis Kit | TaKaRa | Cat # 6110A |
| Tumor Dissociation Kit | Miltenyi Biotec | 130-096-730 |
| Arginase Activity Assay Kit | Sigma | MAK112-1KT |
| Experimental Models: Cell Lines |  |  |
| Mouse melanoma cell line: B16 cells | 3142C0001000000039 | National Infrastrusture of Cell Line Resource |
| Mouse melanoma cell line: B16-OVA cells | This paper | N/A |
| Mouse lung cancer cell line: Lewis lung cancer cells | 3131C0001000800007 | National Infrastrusture of Cell Line Resource |
| Experimental Models: Organisms/Strains |  |  |
| Mouse: C57BL/6J | Charles River Company | 219 |
| Mouse: C57BL/6J–Gpr84/BRL | BRL+ Medicine | KO-0014 |
| Oligonucleotides |  |  |
| PCR Primer Mouse-GAPDH-F: TGGCCTTCCGTGTTCCTAC | This paper | N/A |
| PCR Primer Mouse-GAPDH-R: GAGTTGCTGTTGAAGTCGCA | This paper | N/A |
| PCR Primer Mouse-NCF4-F: GTCATCGAGGTCAAAACAAAAGG | This paper | N/A |
| PCR Primer Mouse-NCF4-R: GCCCATGTAGACTTTGGCTG | This paper | N/A |
| PCR Primer Mouse-ARG1-F: CTCCAAGCCAAAGTCCTTAGAG | This paper | N/A |
| PCR Primer Mouse-ARG1-R: GGAGCTGTCATTAGGGACATCA | This paper | N/A |
| PCR Primer Mouse-CYBB-F: AGTGCGTGTTGCTCGACAA | This paper | N/A |
| PCR Primer Mouse-CYBB-R: GCGGTGTGCAGTGCTATCAT | This paper | N/A |
| PCR Primer Mouse-NOS2-F:  GTTCTCAGCCCAACAATACAAGA | This paper | N/A |
| PCR Primer Mouse-NOS2-R:  GTGGACGGGTCGATGTCAC | This paper | N/A |
| PCR Primer Human-GAPDH-F: GGAGCCAAAAGGGTCATCATCTC | This paper | N/A |
| PCR Primer Human-GAPDH-R: GAGGGGCCATCCACAGTCTTCT | This paper | N/A |
| PCR Primer Human-NCF4-F: CGGGCCGAGAGTGACTTTG | This paper | N/A |
| PCR Primer Human-NCF4-R: TCTTCACCTCGATGACGAAAAC | This paper | N/A |
| PCR Primer Human-ARG1-F: GTGGAAACTTGCATGGACAAC | This paper | N/A |
| PCR Primer Human-ARG1-R: AATCCTGGCACATCGGGAATC | This paper | N/A |
| PCR Primer Human-CYBB-F: ACCGGGTTTATGATATTCCACCT | This paper | N/A |
| PCR Primer Human-CYBB-R: GATTTCGACAGACTGGCAAGA | This paper | N/A |
| PCR Primer Human-NOS2-F:  TTCAGTATCACAACCTCAGCAAG | This paper | N/A |
| PCR Primer Human-NOS2-R:  TGGACCTGCAAGTTAAAATCCC | This paper | N/A |
| Software and Algorithms |  |  |
| Magnetic bead sorter | Miltenyi Biotec | N/A |
| Spectrophotometer | Thermo Fisher Scientific | N/A |
| MACSmix Tube Rotator | Miltenyi Biotec | 130-090-753 |
| ImageJ | Schneider et al., 2012 | https://imagej.nih.gov/ij/ |
| GraphPad Prism software 7.0 | GraphPad Software | https://www.graphpad.com |
| Flow Jo_v10 | FlowJo | http://www.flowjo.com/ |
| Other |  |  |
| Human peripheral blood lymphocyte separation fluid | TBDscience | LTS10771 |
| CD11b MicroBeads, human and mouse | Miltenyi Biotec | No. 130-049-601 |
| Auto MACS Buffer | Miltenyi Biotec | 130-091-221 |
| RNA isoplus | TaKaRa | Cat # 639676 |
| Pierce™ ECL Western Blotting Substrate | Thermo Fisher Scientific | 32109 |
| TWEEN - 80 | Sigma | 59924-100G-F |

Supplementary Text

**Figure. S1. MDSCs increased in esophageal cancer development.** (a) Body weights of mice stimulated with or without 4-NQO were measured during day 106-162 after 4-NQO stimulation (Control, n=4; 4-NQO, n=10). (b) Gross and microscopic specimens of induced esophageal cancer investigated in normal control (Control, n=3), early stage (D113, n=3) and late stage of esophageal cancer (D162, n=7) after 4-NQO stimulation. (c) Percentages of immune cells, including MDSCs, CD4^+^ T, CD8^+^ T, DCs, monocytes/macrophages, B, and NK cells analyzed in esophageal cancer tissues in Control, D113 and D162 after 4-NQO stimulation by flow cytometry. (d) The proportion of G-MDSCs and M-MDSCs were analyzed during esophageal cancer development by flow cytometry. (e-g) Representative images of MDSCs (CD11b^+^CXCR2^+^) and CD8^+^ T cells in tumor site; the total numbers and destiny of MDSCs and CD8^+^ T cells have been calculated by Case Reviewer. (h) The distance of MDSCs and CD8^+^ T cells was also calculated by Case Reviewer. *p < 0.05, **p < 0.01, *** p < 0.001.

**Figure. S2.** **The heatmap of gene expression in the data from GEO.** The cluster analysis shows the genes with increased or decreased expression in esophageal cancer mice, induced MDSCs and colorectal cancer mice respectively. Log2 Fold Change ≥1.5 and P value <0.05 was selected as the statistical difference.

**Figure. S3. The expression patterns of GPR84 in tumor bearing mice.** (a) The percentages and relative expression of GPR84 in immune cells purified from esophageal tumors produced in response to 4-NQO stimulation, including MDSCs, B cells, dendritic cells, macrophages, NK, and T cells analyzed using qPCR and flow cytometry. (b) Percentages of GPR84^+^ cells in immune cells from LLC and B16F0 tumor tissues were analyzed by flow cytometry. **p < 0.01, *** p < 0.001.

**Figure. S4. *GPR84^-/-^* did not affect the percentage and function of immune cells in tumor free mice.** (a)The whole subset of MDSCs (CD11b^+^Gr1^+^) and macrophages (CD11b^+^F4/80^+^) were investigated in the spleen from *WT* and *GPR84^-/-^* tumor free mice. (b-c) The distribution of B cells (B220+), NK cells (CD49b^+^), CD4^+^ T cells (CD3^+^CD4^+^) and CD8^+^T cells (CD3^+^CD8^+^) were detected in *WT* and *GPR84^-/-^* tumor free mice. (d-e) The expression of functional molecule in CD4^+^ T cells (IL-4, IL-10, IL-17A, IL-2) and CD8^+^T cells (IFN-γ, Granzyme-B, Perforin, IL-2 and TNF-α) were compared between *WT* and *GPR84^-/-^* tumor free mice.

**Figure. S5.** **GPR84 blockade inhibited the expression of immunosuppressive molecule.** (a) Relative expression of CYBB, ARG1, NCF4, and iNOS in GPR84-antagonist-treated esophageal cancer tissue-derived MDSCs analyzed using qPCR. (b) IL-10 and TGF-β levels were investigated by ELISA. Relative immunosuppressive molecule expression (c) and ARG1 activity (d) in GPR84-antagonist-treated bone marrow cells in the presence of GM-CSF and G-CSF. * p < 0.05, **p < 0.01.

**Figure. S6. GPR84 deficiency or blockade reduced PD-L1 expression.** (a and b) Volcano plot and histogram showing genes expression in *GPR84^-/-^* spleen MDSCs derived from LLC tumor bearing mice compared with *WT* MDSCs, as determined by RNA-seq (n = 3 mice per group). (c) Principal component analysis (PCA) plot of PD-L1 expression from the RNA-seq data. (d and e) Flow cytometry and immunofluorescence assay were used to examine PD-L1 expression in MDSCs. (f) PD-L1 expression in MDSCs derived from spleens of GPR84^-/-^ and WT B16F0 tumor-bearing mice by flow cytometry. (g) Percentages of PD-L1^+^MDSCs with or without GPR84-antagonist treatment at different times were examined by flow cytometry. * p < 0.05, **p < 0.01.

**Figure. S7. The definition of GPR84 risk.** (a) KEGG pathway enrichment analysis of esophageal squamous cell cancer (ESCC) patients from TCGA were identified into GPR84^high expression^ and GPR84^low expression^ group. Volcano plot (b) and top ten differential genes (c) that changed in RNA-seq results of GPR84^+^ and GPR84^-^ MDSCs from patients with ESCC.

**Figure. S8. The study design of combination therapy.** (a-c) The phonograph showed the treatment of anti-PD-1 or GPR84 antagonist on tumor bearing mice. Proportions of CD8^+^ T cells (d) and CD69, IFN-γ, and Ki67 in CD8^+^ T cells (e) from the spleens of B16 tumor-bearing mice with the corresponding treatment were measured using flow cytometry. * p < 0.05, **p < 0.01, ***p < 0.001.

Figure. S1.

**
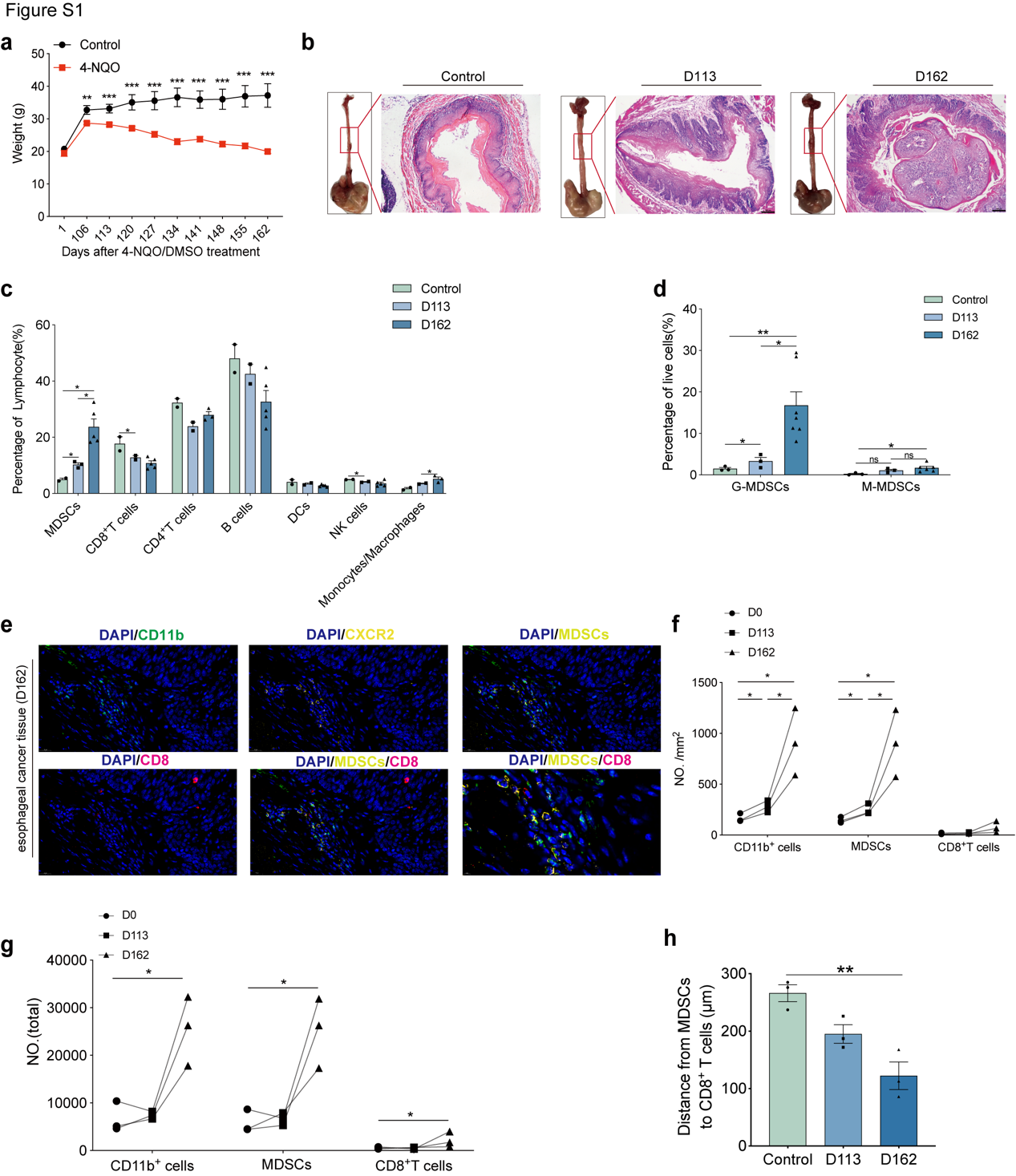
**

**Figure. S1. MDSCs increased in esophageal cancer development.**

Figure. S2.

**
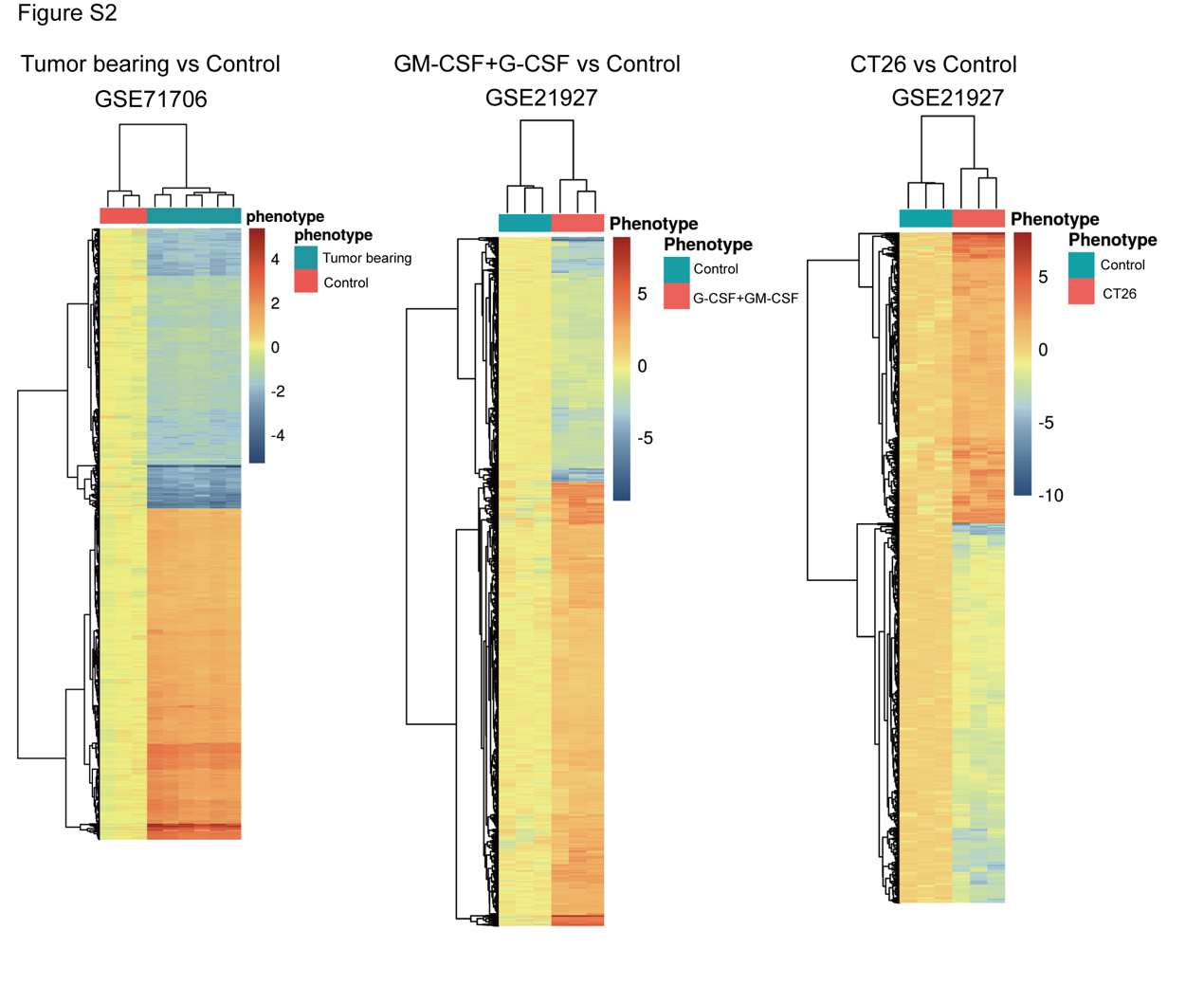
**

**Figure. S2.** **The heatmap of gene expression in the data from GEO.**

Figure. S3.

**
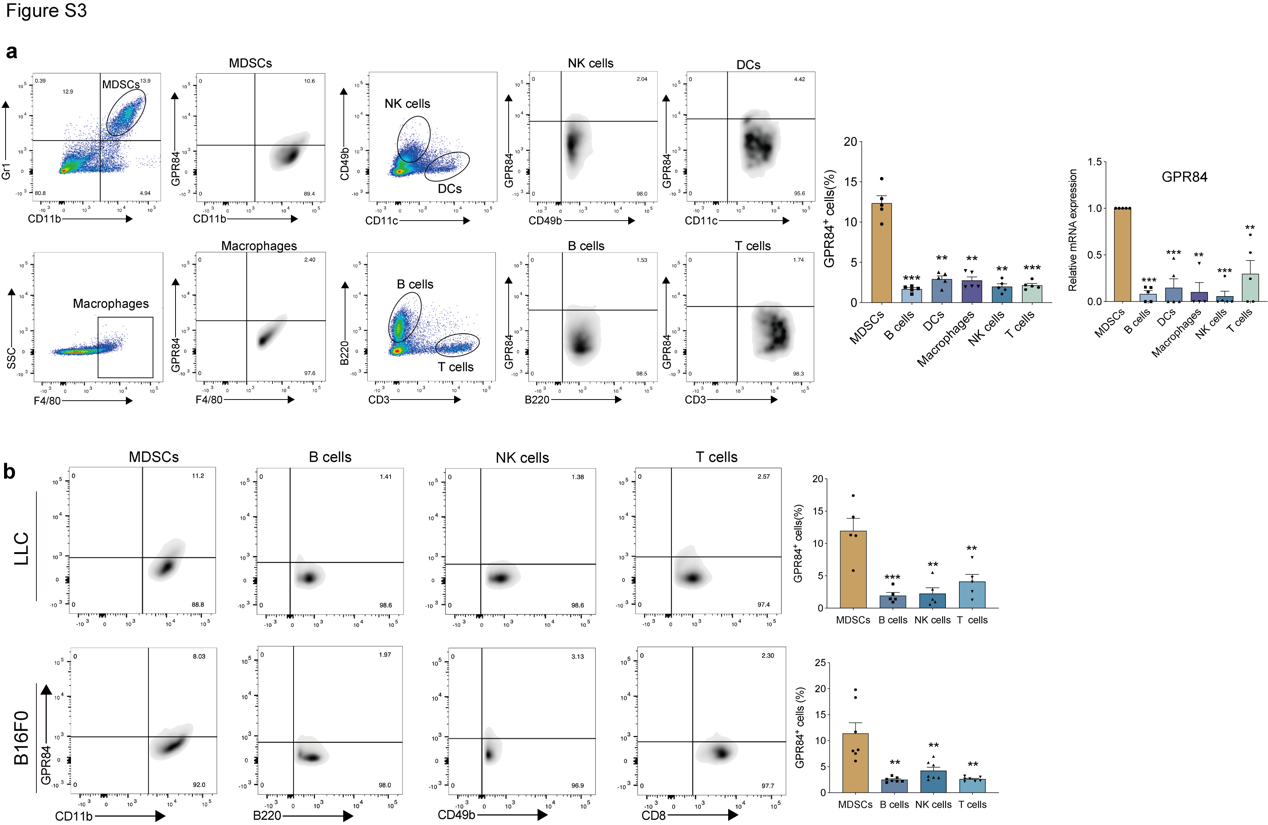
**

**Figure. S3. The expression patterns of GPR84 in tumor bearing mice.**

Figure. S4.


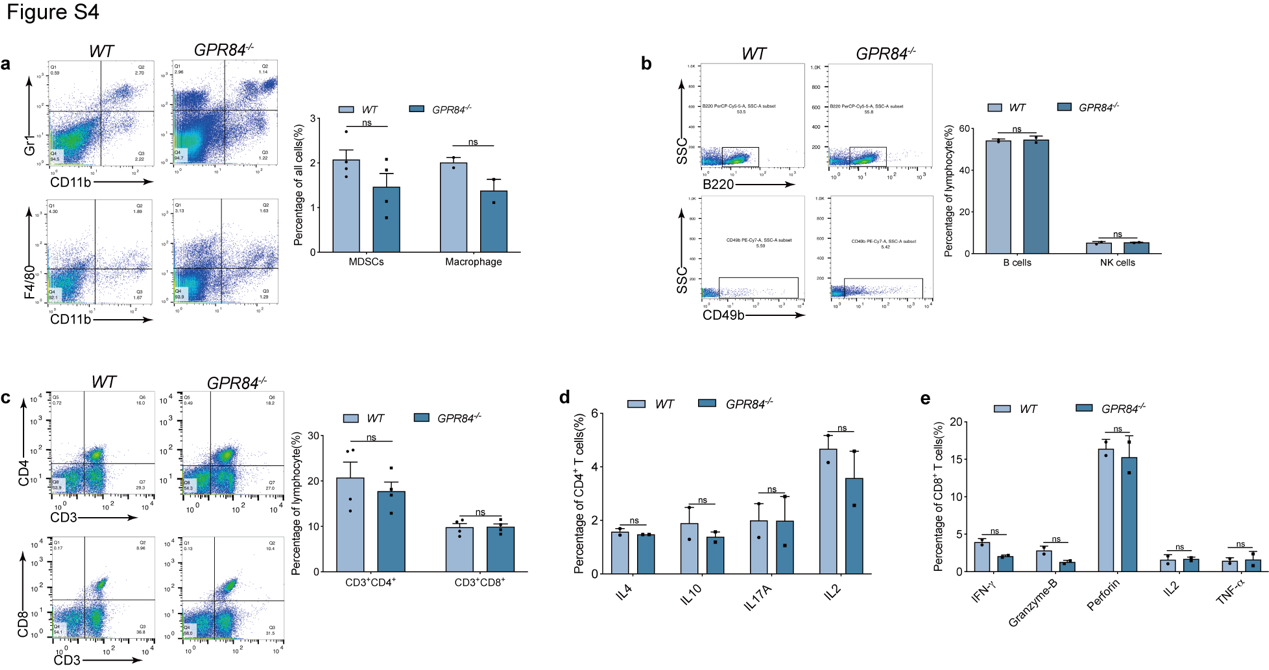


**Figure. S4. *GPR84^-/-^* did not affect the percentage and function of immune cells in tumor free mice.**

Figure. S5.


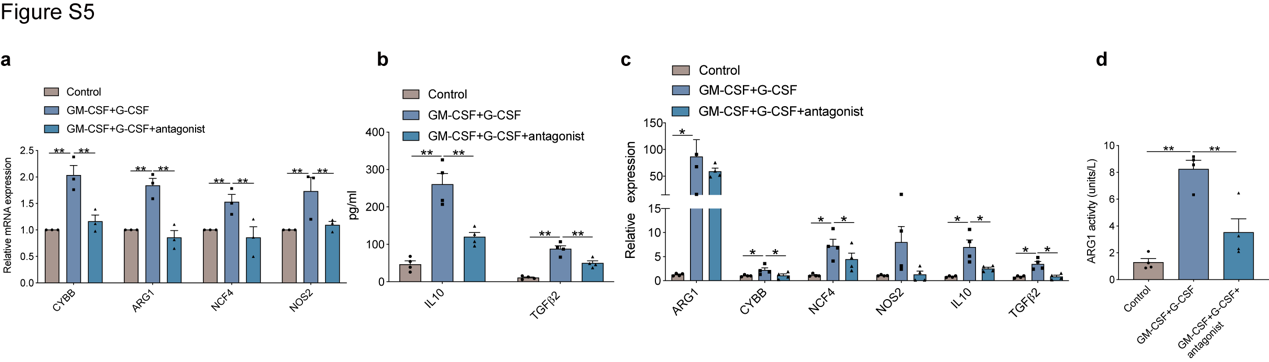


**Figure. S5. GPR84 blockade inhibited the expression of immunosuppressive molecule.**

Figure. S6.


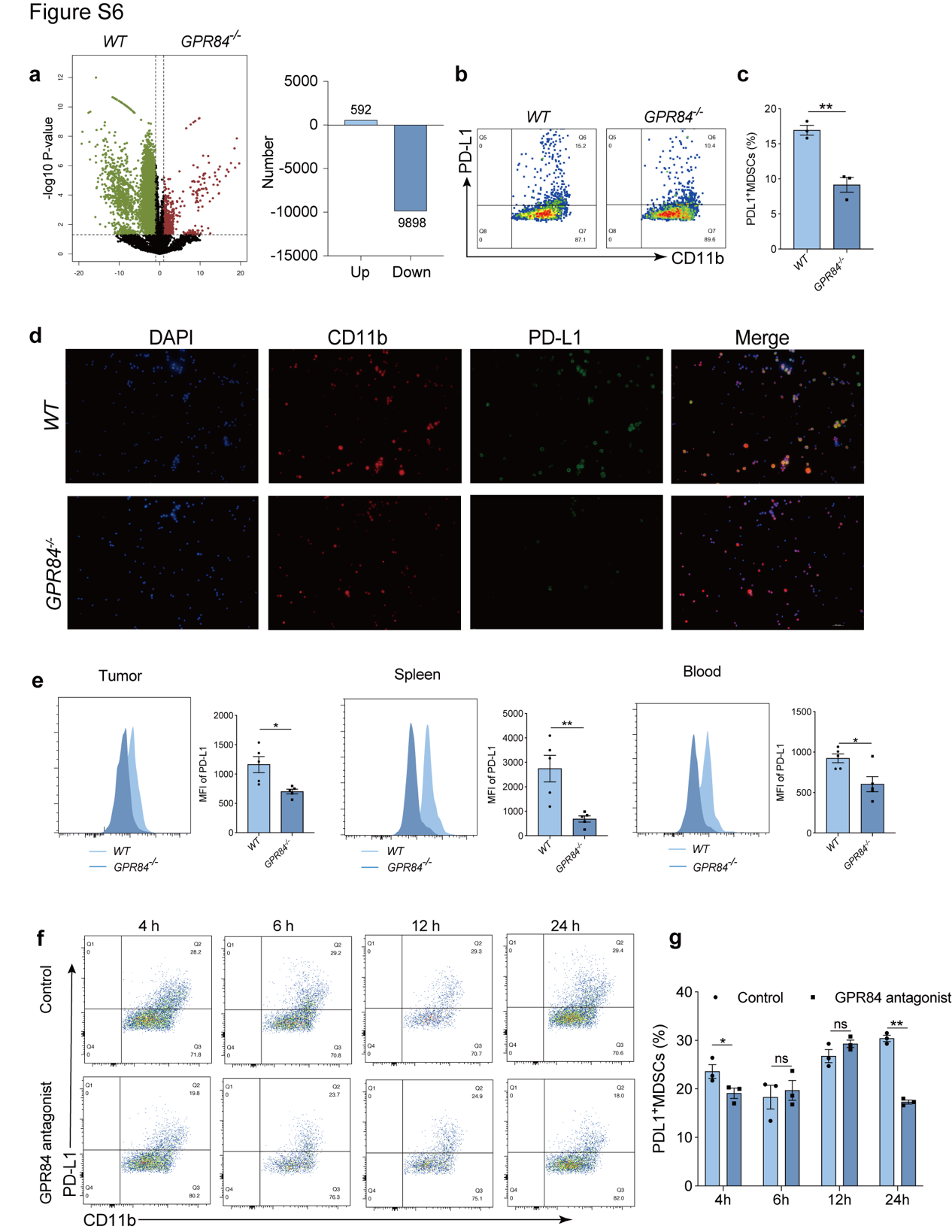


**Figure. S6. GPR84 deficiency or blockade reduced PD-L1 expression.**

Figure. S7.


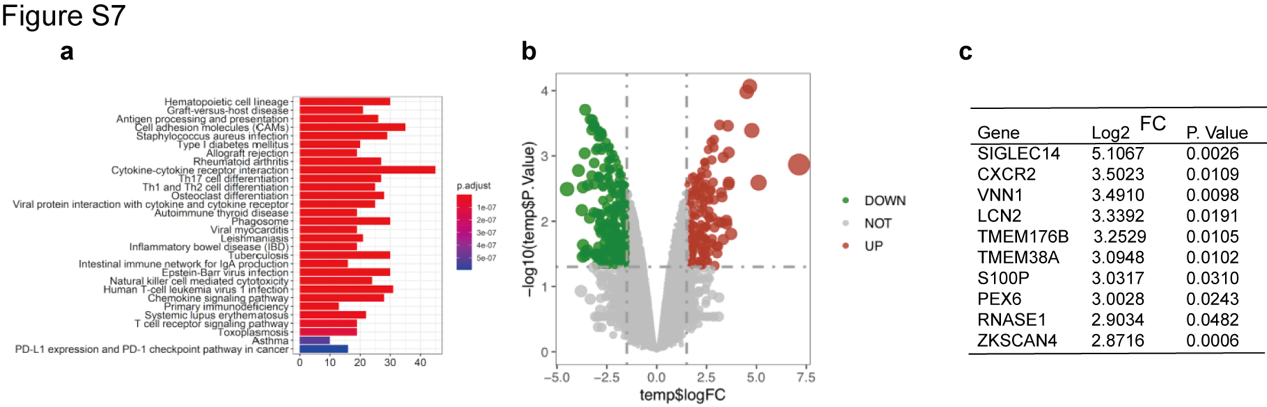


**Figure S7. The definition of GPR84 risk.**

Figure. S8.


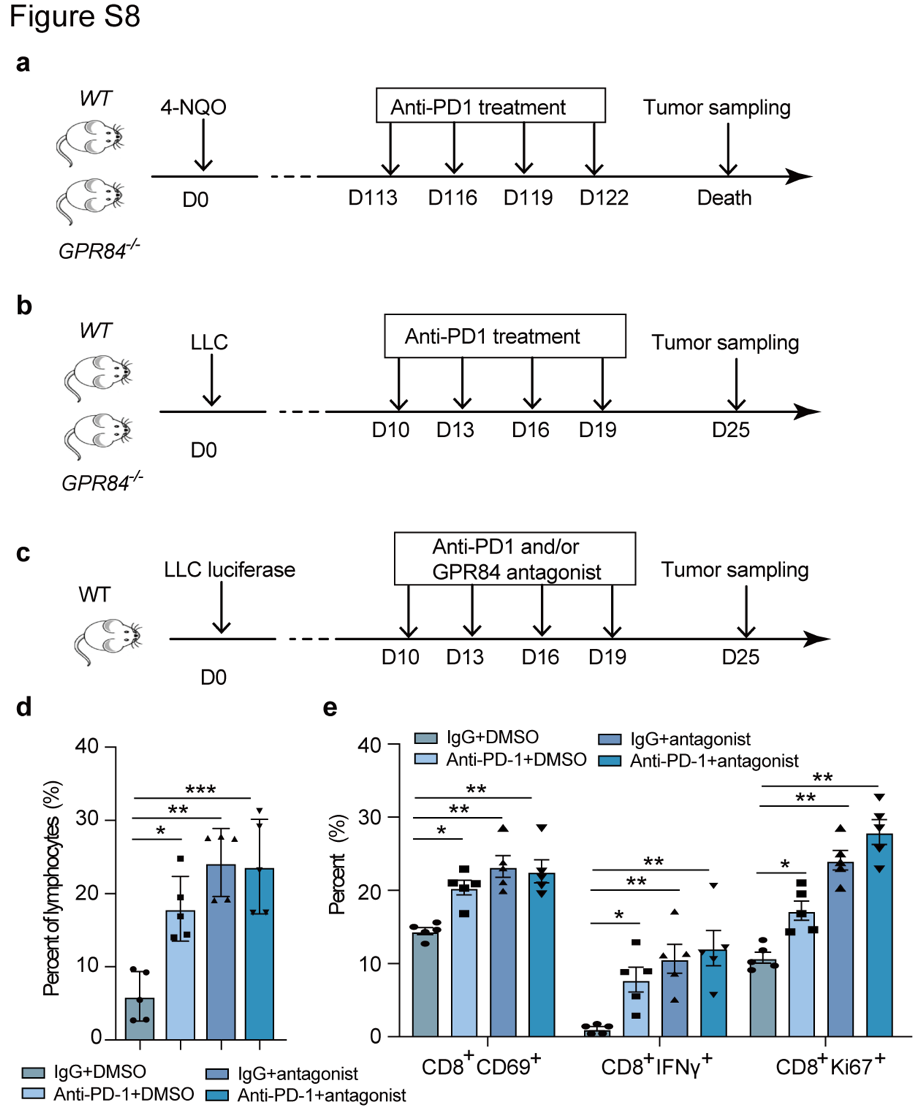


**Figure S8. The study design of combination therapy.**

Table S1.

| CEBPB | GR-b |
| --- | --- |
| GR | RXR-a |
| TFII-I | NF-1 |
| GR-a | HNF-3α |
| ER-a | Pax-5 |
| IRF-2 | XBP-1 |
| YY1 | AP-2αA |
| FOXP3 | PR B |
| TFIID | PR A |

The 17 predicted transcription factors via *PROMO* website.

| Name | Score |
| --- | --- |
| C/EBPβ | 13.7119 |
| FOXP3 | 8.72053 |
| YY1 | 8.19127 |

The binding site score of predicted transcription factors via *JASPAR* website.

Table S2.

| **Patient NO.** | **Response** | **Cancer type** | **Gender** | **Age** | **Stage** | **Smoke** | **Mutation** | **Ki67%** |
| --- | --- | --- | --- | --- | --- | --- | --- | --- |
| P1 | R | NSCLC | Female | 58 | III | No | None | 5-10 |
| P2 | R | NSCLC | Male | 58 | III | Yes | Kras | 70 |
| P3 | R | EAC | Male | 64 | IV | No | None | 60 |
| P4 | R | ESCC | Male | 63 | II | Yes | ND | 80 |
| P5 | R | Cervical carcinoma | Female | 50 | IV | No | ND | ND |
| P6 | R | rectal cancer | Male | 82 | IV | No | ND | ND |
| P7 | NR | NSCLC | Male | 63 | III | Yes | ND |  |
| P8 | R | NSCLC | Male | 66 | II | Yes | None | 70 |
| P9 | R | Cervical  carcinoma | Female | 53 | II | No | ND | ND |
| P10 | R | NSCLC | Male | 67 | II | Yes | EGFR-18 | ND |
| P11 | NR | NSCLC | Female | 54 | II | No | EGFR-18,19,20,21 | 60 |
| P12 | R | Breast cancer | Female | 62 | IV | No | ND | 10 |
| P13 | NR | NSCLC | Male | 61 | II | Yes | None | 15 |
| P14 | R | NSCLC | Female | 64 | II | No | None | ND |
| P15 | NR | NSCLC | Female | 57 | IV | No | RET | 20 |
| P16 | NR | GCA | Male | 71 | III | Yes | None | 70 |
| P17 | R | NSCLC | Male | 61 | IIIA | Yes | MET | ND |
| P18 | NR | NSCLC | Male | 55 | IIIB | Yes | ND | 70 |
| P19 | NR | NSCLC | Male | 61 | IA | Yes | ND | 15 |
| P20 | NR | ESCC | Male | 62 | IV | Yes | ND | ND |
| P21 | R | NSCLC | Male | 64 | IVA | No | None | 80 |
| P22 | R | NSCLC | Female | 64 | IA | No | None | ND |
| P23 | R | NSCLC | Male | 68 |  | Yes | ND | ND |
| P24 | R | NSCLC | Male | 65 | III | Yes | None | 10 |
| P25 | R | Cervical  carcinoma | Female | 60 | IVB | No | ND | 40 |
| P26 | R | NSCLC | Male | 48 | IIIB | Yes | ND | 10 |
| P27 | NR | Synovial sarcoma | Male | 56 | IV | No | ND | 5 |
| P28 | R | melanoma | Male | 61 | IV | Yes | ND | 25 |
| P29 | NR | HCC | Male | 60 |  | Yes | ND | 70 |
| P30 | R | Rectal cancer | Female | 41 | IVC | No | Kras | 90 |
| P31 | R | NSCLC | Female | 65 | IVB | No | ND | 20 |
| P32 | R | NSCLC | Male | 62 | IVA | Yes | UGT1A1 | 70 |
| **Patient NO.** | **Response** | **Cancer type** | **Gender** | **Age** | **Stage** | **Smoke** | **Mutation** | **Ki67%** |
| P33 | NR | NSCLC | Female | 54 | IA | No | None | ND |
| P34 | NR | NSCLC | Female | 54 | IA | No | None | ND |
| P35 | R | NSCLC | Female | 44 | IV | No | None | 5 |
| P36 | R | NHL | Female | 63 | II | No | ND | 90 |
| P37 | R | NSCLC | Male | 63 | IV | No | ND | 50 |
| P38 | NR | pheochromocytoma | Male | 62 | IV | No | ND | 8 |
| P39 | NR | Synovial sarcoma | Male | 43 | IV | No | CTNNB1\TAP1 | 15 |
| P40 | R | NSCLC | Female | 73 | IV | No | LR58R T790M | ND |
| P41 | R | NSCLC | Female | 56 | IV | No | BRAF\V600E | 60 |
| P43 | NR | NSCLC | Male | 58 | IIIB | Yes | ND | 50 |
| P44 | R | NSCLC | Female | 52 | II | No | EGFR | 10 |
| P45 | NR | NSCLC | Male | 55 | IIIB | No | None | ND |
| P46 | R | SCLC | Male | 57 | IV | No | ND | ND |
| P47 | R | Breast cancer | Female | 55 | IV | No | ND | 10 |

**Table S2. Clinical parameters of the patients**
